# The miR395b–ABI5 module regulates amylopectin branching and biosynthesis and affects lotus root quality

**DOI:** 10.1093/plphys/kiaf554

**Published:** 2025-11-05

**Authors:** Fenghua Li, Mengying Tan, Xiaojing Fan, Qianru Niu, Shangjie Chen, Peng Wu, Kai Feng, Shuping Zhao, Liangjun Li

**Affiliations:** School of Horticulture and Landscape Architecture, Yangzhou University, Yangzhou 225009, China; School of Horticulture and Landscape Architecture, Yangzhou University, Yangzhou 225009, China; School of Horticulture and Landscape Architecture, Yangzhou University, Yangzhou 225009, China; School of Horticulture and Landscape Architecture, Yangzhou University, Yangzhou 225009, China; School of Horticulture and Landscape Architecture, Yangzhou University, Yangzhou 225009, China; School of Horticulture and Landscape Architecture, Yangzhou University, Yangzhou 225009, China; School of Horticulture and Landscape Architecture, Yangzhou University, Yangzhou 225009, China; School of Horticulture and Landscape Architecture, Yangzhou University, Yangzhou 225009, China; School of Horticulture and Landscape Architecture, Yangzhou University, Yangzhou 225009, China; Joint International Research Laboratory of Agriculture and Agri-Product Safety of Ministry of Education of China, Yangzhou University, Yangzhou 225009, China

## Abstract

Starch is an important carbohydrate in lotus (*Nelumbo nucifera* Gaertn.) roots, which are edible rhizomes, and starch biosynthesis and metabolism strongly influence lotus root yield and quality. Plant microRNAs (miRNAs) have a significant effect on crop yield, quality, and starch biosynthesis, but the molecular mechanism by which miRNAs regulate starch biosynthesis is unknown. In this study, miR395b expression levels showed significant differences in lotus germplasms with different starch contents. Overexpressing *MIR395b* reduced amylopectin levels and medium- and long-branched chain percentages in lotus roots, decreasing total starch accumulation. Inhibiting miR395b function (using short tandem target mimics) or overexpressing *ABA Insensitive 5* (*NnABI5*) increased amylopectin content and medium- and long-branched chain proportions in lotus roots, significantly enhancing total starch content. miR395b specifically targeted and repressed the expression of the transcription factor NnABI5. Furthermore, NnABI5 promoted amylopectin biosynthesis and medium- and long-branched chain proportions in lotus roots by binding to the promoter region of the amylopectin biosynthesis-related genes *starch synthetase 2* (*NnSS2*) and *starch branching enzyme II* (*NnSBEII*) and activating their expression. Our study reveals the critical role of the miR395b–NnABI5 module in starch biosynthesis in lotus roots, providing a theoretical foundation for lotus root quality and yield improvement.

## Teaser

ABA Insensitive 5 (NnABI5) is an atypical target of miR395b and affects lotus root quality by activating *starch synthetase 2* and *starch branching enzyme II* expression.

## Introduction

Lotus (*Nelumbo nucifera* Gaertn.) is a perennial aquatic herb in the Nelumbonaceae family, and its roots, the edible part of the rhizome, are rich in starch, protein, polysaccharides, polyphenols, antioxidants and mineral elements (Huang et al. 2021; Zhao et al. 2023). The starch content in the roots is an important factor affecting lotus quality (Zhao et al. 2022a). Based on the starch content, lotus can be categorized as powdered or crispy lotus. Powdered lotus has higher total starch and amylopectin contents, making it viscous, delicate, and soft. By contrast, crisp lotus has lower total starch and amylopectin contents (Copeland et al. 2009; Naguleswaran et al. 2014; Goldstein et al. 2016; Zhang et al. 2018). Although lotus is widely cultivated across the globe, little research has been conducted on lotus root quality improvement. Therefore, determining the molecular mechanism by which lotus roots synthesize starch is of great significance for improving lotus root quality.

Starch, including amylose and amylopectin, is formed by the polymerization of glucose molecules. Amylose is synthesized by granule-bound starch synthetase (GBSS), whereas amylopectin is synthesized by starch synthetase (SS), starch branching enzyme (SBE), and debranching enzyme (Seung and Smith 2019; Huang et al. 2021). Among them, multiple SS and SBE isoenzymes play a decisive role in amylopectin chain length and synthesis. In rice grain, *OsSSI* and *OsSSIIIb* synthesize short and long chains of amylopectin, respectively, and in *Arabidopsis* leaves, *AtSS2* extends medium-length glucan chains (Patterson et al. 2018; Morita et al. 2023). Starch granule morphology and amylopectin content are altered after the deletion of the genes encoding *HvSBEs* and *OsSBEIIb* (Regina et al. 2010; Miura et al. 2021; Luo et al. 2022b). In rhizomatous crops such as cassava and potato, the amylopectin content decreased and the amylose content increased after *MeSS2* and *StSBEII* were silenced (Tuncel et al. 2019; He et al. 2022). Our previous study showed that the *NnSS1* gene plays a key regulatory role in the starch quality of lotus roots (Wu et al. 2021). In addition, *NnSBEII* interacts with *NnSS2* to form complex proteins, enhancing the activity of *NnSBEII* enzyme and promoting amylopectin synthesis (Zhao et al. 2025). These studies demonstrate that SS and SBE enzymes are essential for amylopectin biosynthesis.

Starch biosynthesis is influenced by various factors, including microRNAs (miRNAs), transcription factors, and plant hormones. miRNAs are small noncoding RNAs of 18 to 24 nucleotides in length that play important roles in plant growth and development, including plant morphogenesis, signal transduction, biological and abiotic stress tolerance (Rogers and Chen 2013). For example, wheat miR1037 regulates its target gene, a gene coding phosphoglycerate kinase, modulating carbon allocation between starch and sucrose (Meng et al. 2013). miR004-1-5p similarly reduces starch accumulation by suppressing the expression of the sucrose phosphate synthase gene (Lunn and MacRae 2003). miR159 affects starch biosynthesis in maize (*Zea mays*) by negatively regulating the transcription levels of transcription factors ZmMYB138 and ZmMYB115 (Hu et al. 2021). In the miR172/*TOE* module, csi-miR172d downregulation upregulates starch biosynthesis-related genes *CsTOE1.1* and *CsTOE1.2*, significantly decreasing the starch content and number of amyloplasts, which reduces starch accumulation in the callus cells of *Citrus reticulata* (Feng et al. 2022). The miR395 family, which comprises multiple members, is highly conserved in monocotyledonous and dicotyledonous plants (Jones-Rhoades et al. 2006). miR395 regulates crops growth and development by regulating the expression of the ATP sulfase gene *APS* and sulfate transporter *SULTR*, which affects sulfate assimilation and metabolism (Li et al. 2017; Liu et al. 2022; Yang et al. 2022). In apple (*Malus pumila*), miR395 targets WRKY transcription factors to enhance leaf spot disease resistance (Zhang et al. 2017). miR395 is also involved in stress signaling pathways that mediate the interactions between nutrient homeostasis mechanisms (Zhang et al. 2013). These studies demonstrate that miR395 regulates plant growth and development under biotic and abiotic stresses; however, its role in starch biosynthesis has not yet been reported in plants.

The basic leucine zipper (bZIP) transcription factor abscisic acid-insensitive 5 (ABI5) plays a significant role in plant growth and development, biological and abiotic stress responses, and hormone signaling (Skubacz et al. 2016), and regulates seed maturation, dormancy, and germination during seed development (Yu et al. 2015; Ibarra et al. 2016; Yang et al. 2023). It also influences seed growth and development by modulating the expression of *CAT1* and *SHB1* genes, which affects reactive oxygen species homeostasis and embryonic cell division and expansion (Cheng et al. 2014; Bi et al. 2017). Transient overexpressing *ABI5* gene in tomato (*Solanum lycopersicum*) and bananas (*Musa acuminata*) promotes ethylene production and starch and cell wall degradation, accelerating fruit ripening and softening (Song et al. 2022). Although many ABI5 family members have been identified and characterized in plants, few studies have determined their roles in nutritive organ growth, development and quality traits.

Our previous study predicted that miR395b is a key miRNA regulating starch synthesis in lotus roots, and its expression level was significantly negatively correlated with total starch and amylopectin accumulation (Zhu et al. 2022). In this study, we systematically investigated the function of miR395b and revealed that miR395b targeted the downstream transcription factor ABI5 for mRNA cleavage. miR395b upregulation led to the rapid degradation of the NnABI5 transcript, which suppressed the expression of two downstream target genes, *NnSS2* and *NnSBEII*. This suppression reduced amylopectin synthesis, consequently decreasing the total starch content in lotus roots. In summary, our study elucidates the role of the miR395b–NnABI5 module in the amylopectin synthesis pathway in lotus roots, providing theoretical insights for breeding high-quality lotus germplasm.

## Results

### miR395b is associated with starch synthesis in lotus roots

**Figure 1.**
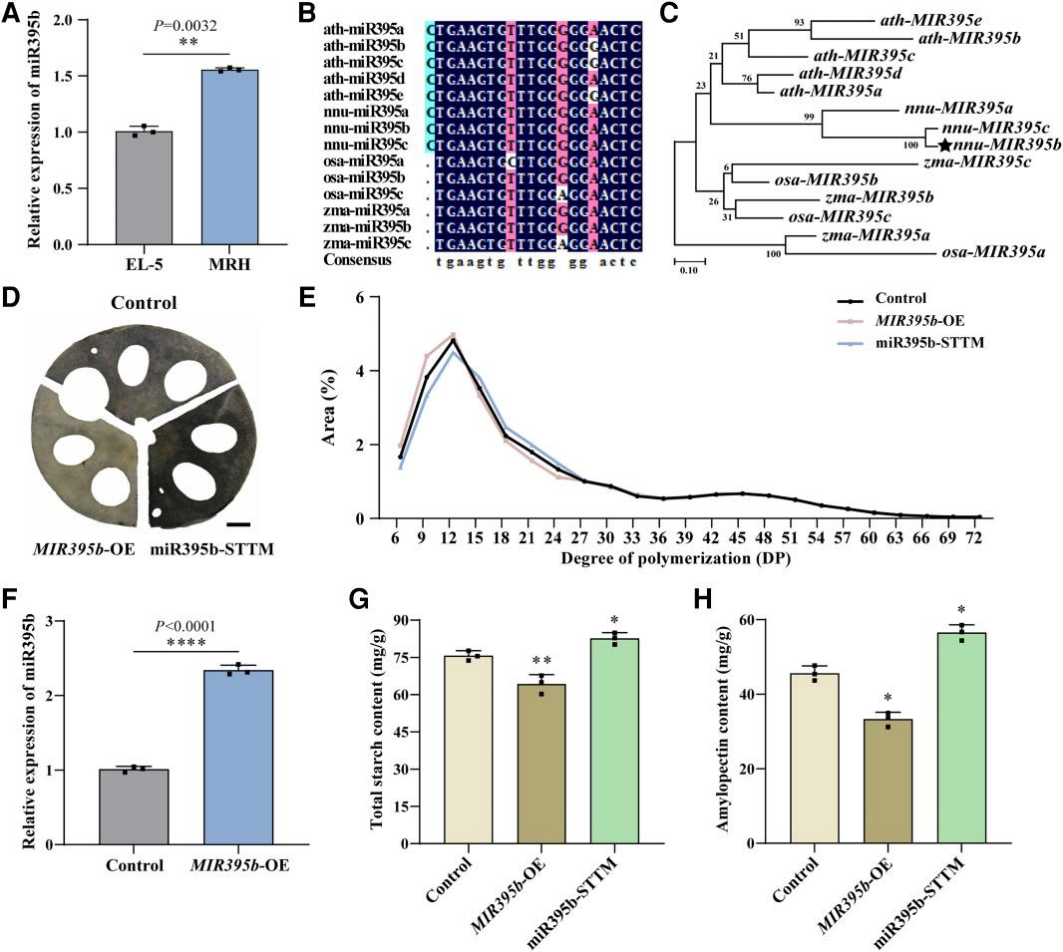
miR395b regulates starch biosynthesis in lotus roots. **A)** RT-qPCR of miR395b levels in developing lotus roots of “EL-5” (high starch content) and “MRH” (low starch content). **B)** Alignment analysis of mature sequences of miR395. The bases with blue, red, and green backgrounds respectively represents 100%, 80%, and 50% of identity. **C)** Phylogenetic analysis of *nnu-MIR395* with other precursors of *MIR395*. The phylogenetic tree was constructed using MEGA software with the maximum likelihood algorithm. Bootstrap values indicate the confidence of each branch, and the scale indicates the branch length. The scale bar represents 0.10 substitutions per site. **D)** Starch detection by iodine staining in lotus roots. The scale bar represents 1 cm. Images were digitally extracted for comparison. The experiments were performed independently twice with similar results, and one representative result is shown. **E)** Differences in the distribution of amylopectin chain lengths between the control, *MIR395b*-OE, and miR395b-STTM. OE: overexpression, STTM: short tandem target mimics. Data are shown as means ± SD (*n* = 3). **F)** RT-qPCR analysis of miR395b levels in lotus roots of the control and *MIR395b*-OE. Data are shown as means ± SD (*n* = 3). **G** and **H)** Starch content in lotus roots of the control, *MIR395b*-OE, and miR395b-STTM. **G)** Total starch content and **H)** amylopectin content. Data are shown as means ± SD (*n* = 3). Statistical analysis was performed using a one-way ANOVA, which is applicable to all statistical analyses in this figure. Significant levels are denoted by asterisks (two-sided Student's *t*-test; *P < 0.05; **P < 0.01; ****P < 0.0001).

To understand the mechanism underlying starch synthesis in lotus roots, we previously conducted integrated analysis of short RNAs and transcripts from developing lotus roots of the varieties “EL-5” (high starch content) and “MRH” (low starch content) (Zhu et al. 2022). We found that miR395b was highly abundant in “MRH” and low in “EL-5,” and the reverse transcription quantitative PCR (RT-qPCR) confirmed the pattern of miR395b abundance in lotus roots (Fig. 1A). Three miR395 family members (nnu-miR395a, nnu-miR395b, nnu-miR395c) in lotus roots were identified using “the Plant MicroRNA Encyclopedia database” (https://www.pmiren.com). Multiple sequence alignment revealed that the mature sequences of miR395 were highly conserved among plant species (Fig. 1B). Based on a comparison of the miR395 precursor sequences in different plants, we found that the sequences were not conserved, except for the mature sequences (Supplementary Fig. S1A). The phylogenetic relationships of *MIR395s* showed that the *nnu-MIR395a/b/c* precursor sequences had high similarity to *Arabidopsis MIR395a* (*ath-MIR159a*) (Fig. 1C). *nnu-MIR395a/b* had a typical stem-loop structure, and the mature sequence was derived from the 5′ arm of the stem-loop structure (Supplementary Fig. S1B). The predicted minimum free energy of miRNA precursors indicated that *nnu-MIR395b* had the highest stability at 46.25 kcal/mol (Supplementary Table S1). In summary, nnu-miR395b is more conserved among plants than the other two differentially expressed miRNAs; thus, we selected nnu-miR395b for subsequent functional studies.

To investigate the function of miR395b in lotus roots, we achieved overexpression and functional inhibition of miR395b using the transgenic and the short tandem target mimic (STTM) methods, respectively, and tested the expression of miR395b by RT-qPCR (Supplementary Fig. S1C and Fig. 1F). We observed the changes in starch content in lotus root tissue through iodine–potassium iodide (I_2_–KI) staining. *MIR395b*-OE had lighter iodine staining, indicating its lower starch content, while miR395b-STTM had darker iodine staining, appearing dark brown (Fig. 1D). We measured the total starch and amylopectin contents of *MIR395b*-OE, miR395b-STTM, and the control. The total starch and amylopectin contents of *MIR395b*-OE decreased by 15% and 27%, respectively, compared with the control, while those of miR395b-STTM increased by 9% and 24%, respectively (Fig. 1, G and H). Ion chromatography was used to analyze the distribution of starch chain lengths in lotus roots and revealed that the chain length distribution in *MIR395b*-OE and miR395b-STTM materials was significantly different from that in the control group. In *MIR395b*-OE, the proportion of short chains with a polymerization degree (DP) of 6 to 12 increased significantly, while the proportion of medium- and long-branched chains (DP 12 to 24) decreased significantly. The proportion of short- and medium- branched chains (DP 6 to 12) was significantly lower in miR395b-STTM than in the control, while the proportion of medium- and long-branched chains (DP 12 to 24) was significantly higher than that in the control (Fig. 1E). These results indicate that miR395b negatively regulates total starch and amylopectin biosynthesis in lotus roots and affects the amylopectin chain length.

**Figure 2.**
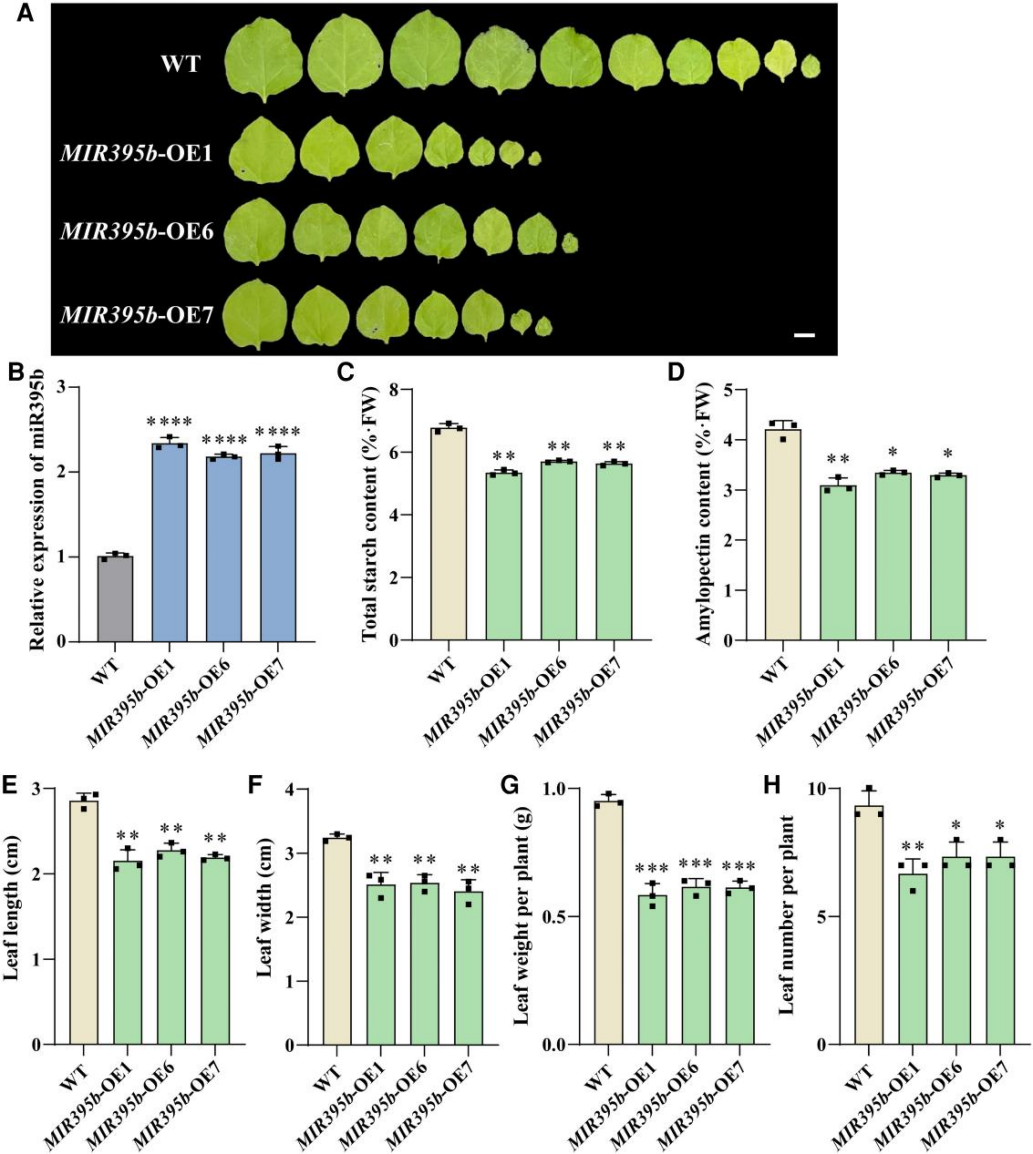
miR395b overexpression inhibits the growth of transgenic tobacco plants. **A)** Phenotypic analysis of tobacco leaves overexpressing miR395b. The scale bar represents 1 cm. Images were digitally extracted for comparison. **B)** RT-qPCR analysis of miR395b levels in leaves of WT and *MIR395b*-OE tobaccos. Data are shown as means ± SD (*n* = 3). **C** and **D)** Total starch and amylopectin contents in rosette leaves at 30 d after planting in wild type and *MIR395b*-OE. Data are shown as means ± SD (*n* = 3). Leaf length **(E)**, leaf width **(F)**, leaf weight **(G)**, and leaf number **(H)** of rosette leaves at 30 d after planting in the wild type and *MIR395b*-OE. Data are shown as means ± SD (*n* = 3). Statistical analysis was performed using a one-way ANOVA, which is applicable to all statistical analyses in this figure. Significant levels are denoted by asterisks (two-sided Student's *t*-test; *P < 0.05; **P < 0.01; ***P < 0.001; ****P < 0.0001).

We overexpressed nnu-miR395b in tobacco (*Nicotiana benthamiana*) and obtained T_3_ generation transgenic tobacco (Fig. 2A). Positive PCR validation and RT-qPCR analysis of the transgenic strains demonstrated that miR395b accumulates in transgenic tobacco (Fig. 2B and Supplementary Fig. S2A). *MIR395b*-OE leaves showed an 18% and 23% decrease in total starch and amylopectin contents, respectively, compared with the wild type (WT) (Fig. 2, C and D). The leaf length and width of *MIR395b*-OE were reduced by 22% and 27%, respectively, compared with WT. Both the weight and number of individual leaves were significantly lower than those in WT (Fig. 2, E to H). These results indicated that miR395b affects the starch properties of tobacco leaves and reduces their biomass.

Further analysis of the tobacco transcriptome data from WT and *MIR395b*-OE revealed 1,021 upregulated and 2,268 downregulated genes (Supplementary Fig. S2B). Kyoto Encyclopedia of Genes and Genomes (KEGG) pathway enrichment scatter plot analysis showed that these differentially expressed genes (DEGs) were primarily involved in plant hormone signal transduction and starch and sucrose metabolism pathways (Supplementary Fig. S2C). Further analysis revealed that the downregulated genes, indicating *NtTIRs*, *NtARFs*, *NtAUXs*, *NtIAAs*, *NtGH3*, and *ABA 8*′*-hydroxylase*, were associated with the synthesis and transduction of the hormones auxin and abscisic acid (ABA) (Supplementary Fig. S3 and Supplementary Table S2). In conclusion, miR395b may influence starch biosynthesis and accumulation and plant yield by regulating the expression of genes related to the auxin and ABA pathways.

### miR395b targets and represses the expression of NnABI5

**Figure 3.**
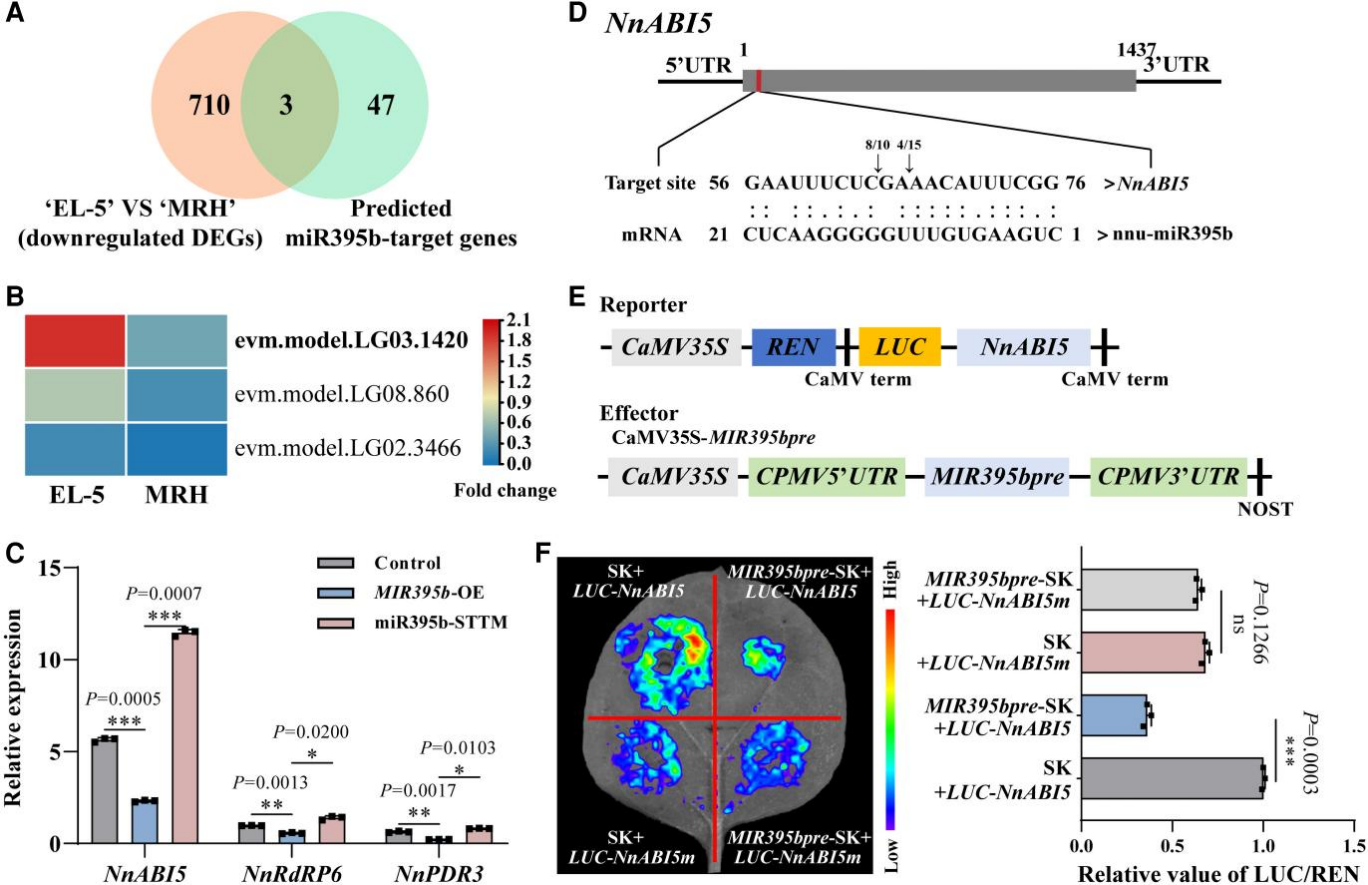
miR395 targets and regulates the expression of *NnABI5*. **A)** Venn diagram showing the extent of overlap between differentially expressed genes (DEGs) in lotus roots of “EL-5” compared with “MRH” (absolute Log_2_[fold change] ≥ 1 and *q*-value ≤ 0.05) and predicted miR395b-target genes. **B)** Heatmap representation of transcript levels for possible miR395b target genes in lotus roots of “EL-5” compared with “MRH”. **C)** RT-qPCR was performed to determine the expression levels of *NnABI5*, *NnRdRP6*, and *NnPDR3* genes in lotus roots of the control, *MIR395b*-OE, and miR395b-STTM. Data are shown as means ± SD (*n* = 3). **D)** 5′ RLM-RACE validation of miR395b target genes. Red box, predicted cleavage site. The arrows indicate the cleavage sites. The numbers above the arrows refer to the number of clones that detected the cleavage site and the total number of clones in the 5′ RLM-RACE assay. Dots represent Watson-Crick pairing. **E)** Effector and reporter constructs used in the tobacco transient expression assay are shown schematically. **F)** Left, dual-LUC activity assay shows that miR395b inhibits NnABI5 transcription. Right, quantitative analysis. Data are shown as means ± SD (*n* = 3). The experiments were performed independently twice with similar results, and one representative result is shown. Statistical analysis was performed using a one-way ANOVA, which is applicable to all statistical analyses in this figure. Significant levels are denoted by asterisks (two-sided Student's *t*-test; *P < 0.05; **P < 0.01; ***P < 0.001; ns, no significant difference).

To explore the network by which miR395b rugulates starch synthesis in lotus roots, we compared the transcriptomes of “EL-5” and “MRH”. We identified 2,112 DEGs, of which 713 were downregulated and 1,399 were upregulated (Supplementary Fig. S4A). KEGG pathway enrichment showed that these DEGs were mainly enriched in metabolic pathways (Supplementary Fig. S4B). Using transcriptome analysis, 50 genes were predicted to be target genes of miR395b, of which 3 had an expression pattern opposite to that of miR395b (Fig. 3A). Notably, *abscisic acid-insensitive 5* (*NnABI5*, evm.model.LG03.1420) had the highest expression level in “EL-5,” and it was significantly higher in “EL-5” than in “MRH,” which was opposite to the pattern observed for miR395b abundance (Fig. 3B). The *NnABI5* expression level was higher compared to *RNA-dependent RNA polymerase 6* (*NnRdRP6*, evm.model.LG08.860) and *pleiotropic drug resistance 3* (*NnPDR3*, evm.model.LG02.3466), as confirmed by RT-qPCR (Fig. 3C). After transient overexpressed miR395b, the *NnABI5* expression decreased significantly, but transient silenced miR395b led the *NnABI5* expression increased significantly. The changes of *NnRdRP6* and *NnPDR3* expression levels were not as significant as *NnABI5* (Fig. 3C). These results suggest that NnABI5 may be involved in starch biosynthesis and regulated by miR395b in lotus roots.

Target gene prediction was performed using the online website psRNATarget to further clarify the miR395b target gene, and the results showed that the NnABI5 coding sequence (CDS) contained the miR395b binding site (Supplementary Fig. S5A). We performed 5′ RNA ligase-mediated rapid amplification of cDNA ends (RLM-RACE) to detect miR395b-dependent cleavage of NnABI5 transcripts in lotus roots. As shown in Fig. 3D, we mainly detected the presence of cleavage sites at 9 to 10th and 11 to 12th nucleotides within the region complementary to miR395b. We generated *LUC-NnABI5* and *LUC-NnABI5m* constructs by cloning the CDS of the firefly luciferase gene (*LUC*) in-frame with an intact (for *LUC-NnABI5*) or mutated miR395b-target region (for *LUC-NnABI5m*). We then coinfiltrated each reporter construct into tobacco leaves with an effector construct cloning the miR395b precursor (*MIR395bpre*-SK) (Fig. 3E and Supplementary Fig. S5B). We coinfiltrated different reporter and effector combinations into tobacco leaves and measured the firefly luciferase activity to *Renilla* luciferase activity (LUC/REN) ratio. The *MIR395bpre*-SK+*LUC-NnABI5* group exhibited reduced LUC activity compared with the empty effector construct and *LUC-NnABI5* group. Coinoculation of *MIR395bpre*-SK with *LUC-NnABI5* did not affect the fluorescence intensity of *LUC-NnABI5m* (Fig. 3F). These results suggest that *NnABI5* is one of the target genes of miR395b and that miR395b regulates starch biosynthesis in lotus roots by repressing NnABI5 transcription.

### The miR395b–NnABI5 module regulates starch synthesis in lotus roots

We analyzed the amino acid sequence of the protein encoded by *NnABI5* using the SMART online analysis website to investigate the regulatory function of NnABI5, found that amino acids 386 to 451 kDa contained a basic region leucin zipper structural domain typical of the bZIP family, indicating that NnABI5 is a bZIP transcription factor (Supplementary Fig. S5C). The homologous sequences of lotus NnABI5 in other plants were searched using BLAST on the NCBI online website, and a phylogenetic tree was produced using MEGA11 software. Lotus NnABI5, *Solanum tuberosum* StABI5 (XM_006341186.2), *Arabidopsis thaliana* AtABI5 (NP_001324735.1), and *Z. mays* ZmABI5 (NP_001048225.1) were closest in affinity; thus, the functions of NnABI5 may be similar to those of homologous genes in potato, *Arabidopsis*, and maize (Supplementary Fig. S5D). Amino acid sequence comparison using DNAMAN software and protein conserved structural domain prediction analysis on the Pfam online website showed that all ABI5 proteins contained typical bZIP structural domains (Supplementary Fig. S6, A and B).

**Figure 4.**
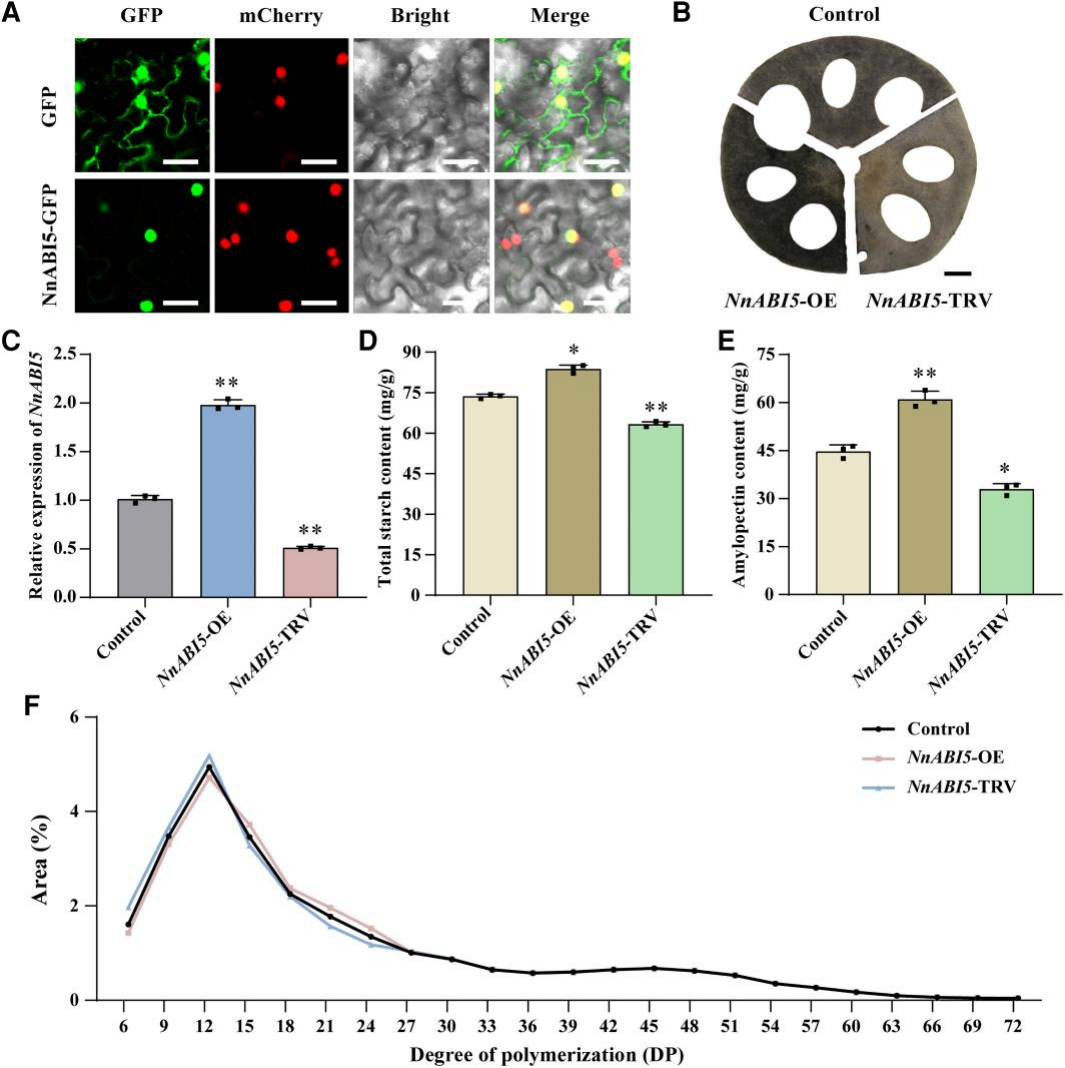
NnABI5 regulates starch biosynthesis in lotus roots. **A)** Subcellular localization of NnABI5 in tobacco leaves. NnABI5-GFP was coinfiltrated with mCherry (nuclear marker) into tobacco leaves. Scale bars, 50 *μ*m. **B)** Starch detection by iodine staining in lotus roots. The scale bar represents 1 cm. Images were digitally extracted for comparison. The experiments were performed independently twice with similar results, and one representative result is shown. **C)** RT-qPCR analysis of NnABI5 levels in lotus roots of the control, *NnABI5*-OE, and *NnABI5*-TRV. Data are shown as means ± SD (*n* = 3). **D** and **E)** Starch content in lotus roots of the control, *NnABI5*-OE, and *NnABI5*-TRV. **D)** Total starch content and **(E)** amylopectin content. Statistical analysis was performed using a one-way ANOVA. Data are shown as means ± SD (*n* = 3). Statistical analysis was performed using a one-way ANOVA, which is applicable to all statistical analyses in this figure. Significant levels are denoted by asterisks (two-sided Student's *t*-test; *P < 0.05; **P < 0.01). **F)** Differences in the distribution of amylopectin chain lengths among the control, *NnABI5*-OE, and *NnABI5*-TRV. Data are shown as means ± SD (*n* = 3).

To determine the subcellular localization of NnABI5, we transiently expressed the green fluorescent protein (GFP)-fused NnABI5 protein in tobacco, with nuclear localization marker proteins *p35S:GFP* and mCherry as negative and positive controls. Confocal microscopy revealed that the NnABI5-GFP fusion protein signal was present in the nucleus, indicating that NnABI5 localized in the nucleus (Fig. 4A). To determine the role of NnABI5 in starch synthesis in lotus roots, we increased the expression level of NnABI5 using the pCAMBIA1300 expression vector. We also selected a gene-specific region of NnABI5 to generate the *NnABI5*-TRV structure and silence NnABI5 (Supplementary Fig. S6C). RT-qPCR showed *NnABI5* expression increased after overexpression and decreased after silencing (Fig. 4C). I_2_–KI staining showed that *NnABI5*-OE exhibited darker iodine staining, indicating a higher starch content, whereas *NnABI5*-TRV showed a lighter iodine staining (Fig. 4B). Compared with the control, the total starch content of *NnABI5*-OE increased by 14%, and the amylopectin content increased by 33%. The total starch content of *NnABI5*-TRV decreased by 16%, and the amylopectin content decreased by 27% (Fig. 4, D and E). The amylopectin chain length distribution showed that the proportion of short chains with a DP of 6 to 12 decreased significantly in *NnABI5*-OE, whereas the proportion of medium and long chains with a DP of 12 to 24 increased significantly (Fig. 4F). The proportion of short- and medium-branched chains (DP 6 to 12) in *NnABI5*-TRV was significantly higher than that in the control, increasing by 8%. The proportion of medium- and long-branched chains (DP 12 to 24) was significantly lower than that in the control, decreasing by 7% (Fig. 4F). These results indicate that NnABI5 positively regulates total starch and amylopectin biosynthesis, and influences the amylopectin chain length distribution in lotus roots.

We transiently overexpressed *MIR395b* in the transient overexpression background of *NnABI5* to verify that miR395b participated the starch synthesis in lotus roots by regulating the transcriptional level of NnABI5 (Supplementary Fig. S7). After iodine staining, *MIR395b*-OE+*NnABI5*-OE exhibited a dark gray color, similar to the control. By contrast, *MIR395b*-OE appeared light gray, and *NnABI5*-OE appeared black (Supplementary Fig. S7A). The expression level of *NnABI5* in the cotransgenic lotus roots of *MIR395b*-OE+*NnABI5*-OE was similar to the control but in contrary to the upregulated expression in *NnABI5*-OE lotus roots (Supplementary Fig. S7C). The amylopectin chain length distribution of *MIR395b*-OE+*NnABI5*-OE was also similar to the control, with no significant differences in the total starch and amylopectin contents. However, both were significantly lower than those in *NnABI5*-OE (Supplementary Fig. S7, B, D, and E). In summary, miR395b in lotus roots downregulates the NnABI5 target gene, thereby inhibiting amylopectin biosynthesis, the amylopectin chain length distribution, and total starch accumulation.

### NnABI5 directly binds to the *NnSS2* and *NnSBEII* promoters and activates their expression

To determine whether NnABI5 is self-activated, the pGBKT7 empty vector and pGBKT7-NnABI5 plasmid were transferred into yeast cells using a yeast two-hybrid (Y2H) system, and the cells were plated on SD/–Trp, SD/–Trp–His, and SD/–Trp–Ade yeast media. The pGBKT7 empty vector grew normally on SD/–Trp-deficient yeast medium but not on SD/–Trp–His or SD/–Trp–Ade yeast medium. The NnABI5 experimental group grew normally on all three defective yeast media (Supplementary Fig. S7F). These results indicate that the NnABI5 transcription factor is self-activated in yeast, suggesting that NnABI5 independently regulates the transcription of its downstream target genes without other genes.

**Figure 5.**
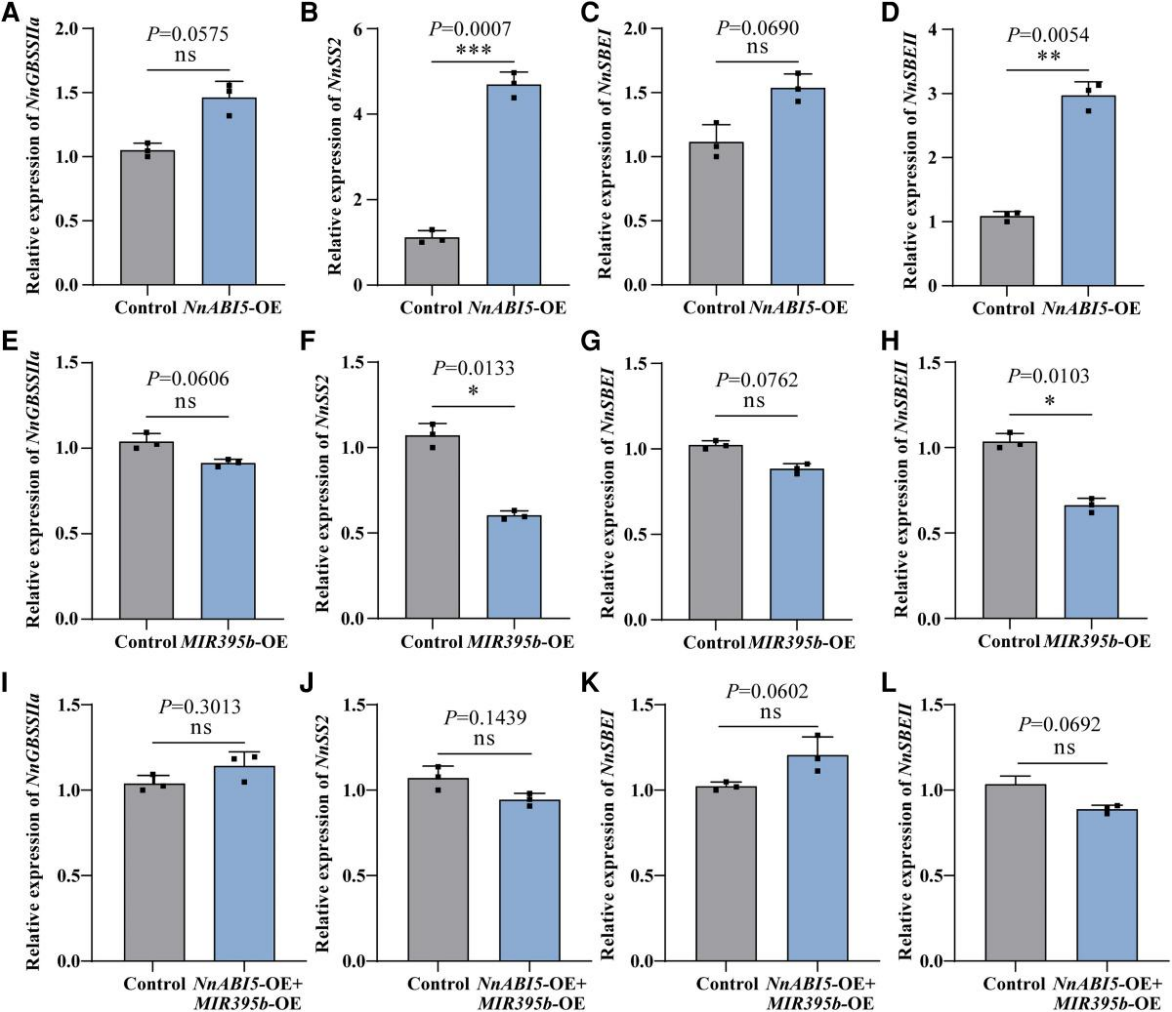
The miR395b–NnABI5 module regulates the expression of starch biosynthesis-related genes in lotus roots. **A** to **D)** RT-qPCR of the transcript levels of starch biosynthesis-related genes in the control and *NnABI5*-OE. Data are shown as mean ± SD (*n* = 3). **E** to **H)** RT-qPCR of the transcript levels of starch biosynthesis-related genes in the control and *MIR395b*-OE. Data are shown as mean ± SD (*n* = 3). **I** to **L)** RT-qPCR of the transcript levels of starch biosynthesis-related genes in the control and *NnABI5*-OE+*MIR395b*-OE. Data are shown as means ± SD (*n* = 3). Statistical analysis was performed using a one-way ANOVA, which is applicable to all statistical analyses in this figure. Significant levels are denoted by asterisks (two-sided Student's *t*-test; *P < 0.05; **P < 0.01; ***P < 0.001; ns, no significant difference).

ABI5 has been shown to directly binds to the ABRE motif (5′-CACGTG-3′) in the promoter regions of downstream genes, promoting gene expression (Carles et al. 2002; Chen et al. 2024a). We obtained a 2,000-bp promoter fragment upstream of lotus starch biosynthesis-related genes to screen for key genes regulating starch synthesis downstream of NnABI5 and found that the promoter regions of only four genes—*NnGBSSIIa*, *NnSS2*, *NnSBEⅠ*, and *NnSBEⅡ*—contained ABRE motifs. We examined the expression levels of these genes in lotus roots after *NnABI5* overexpression using RT-qPCR. *NnSS2* and *NnSBEII* expression levels in *NnABI5*-OE were significantly higher than those in the control, but there were no significant differences in *NnGBSSIIa* and *NnSBEⅠ* expression levels (Fig. 5, A to D). We also examined the expression of these four genes in lotus roots after *MIR395b* overexpression. Increasing the abundance of miR395b significantly decreased *NnSS2* and *NnSBEII* expression levels compared with the control, but there were no significant differences in *NnGBSSIIa* and *NnSBEⅠ* expression levels, which was opposite to the expression trend observed in *NnABI5*-OE (Fig. 5, E to H). To determine whether miR395b participates in this process by regulating the NnABI5 transcription factor, we evaluated the expression levels of four starch biosynthesis-related genes after the cooverexpressing *MIR395b* and *NnABI5*. *NnGBSSIIa* and *NnSBEⅠ* expression levels increased, whereas those of *NnSS2* and *NnSBEII* decreased in *NnABI5*-OE+*MIR395b*-OE lotus roots; however, these changes did not reach a significant level (Fig. 5, I to L). These results suggest that miR395b-dependent NnABI5 play a negative regulatory role in amylopectin synthesis and accumulation in lotus roots. miR395b inhibited the expression of starch synthesis genes by partially suppressing NnABI5 transcription, thereby inhibiting starch synthesis in lotus roots. Moreover, the miR395b–NnABI5 module may play a role in amylopectin synthesis by affecting the expression of the *NnSS2* and *NnSBEII* genes.

**Figure 6.**
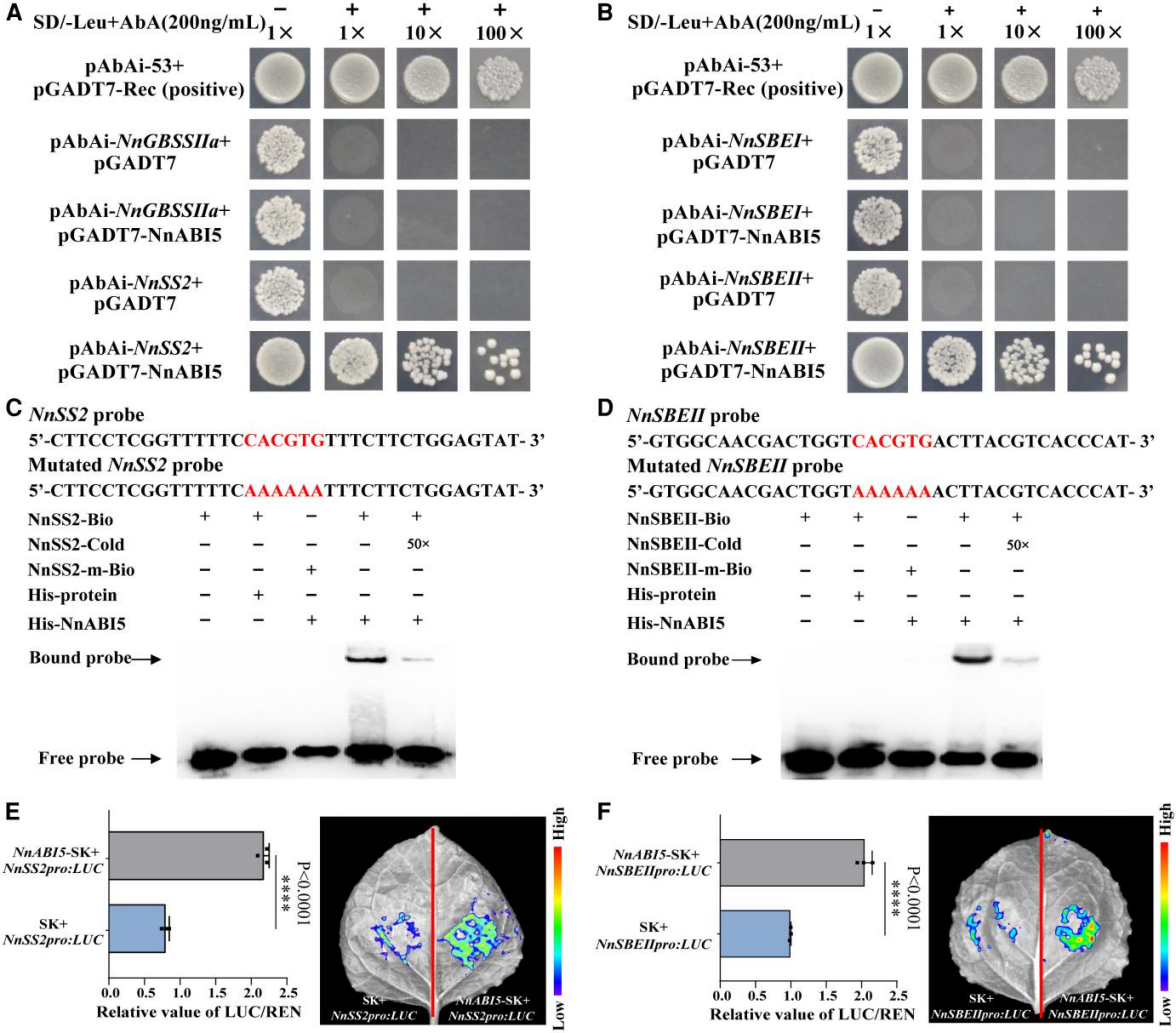
NnABI5 controls the starch biosynthesis in lotus roots. **A** and **B)** Yeast one-hybrid (Y1H) assay showing NnABI5 binding to the *NnSS2* and *NnSBEII* promoters. The positive controls in **(A)** and **(B)** have been reused for the same treatment. “+” and “−” indicate synthetic defined (SD) medium lacking Leu with (+) or without (−) 200 ng/mL AbA. 1×, 10×, and 100× indicate the dilution factor of the yeast cultures before spotting onto the indicated medium (1, 10, and 100 times). **C** and **D)** EMSA was performed to detect the binding of NnABI5 to the *NnSS2* and *NnSBEII* promoters. Red letters represent the binding motifs, and their corresponding mutation motifs. **E)** Left, quantitative analysis. Right, dual-LUC activity assay shows that NnABI5 activates the expression of *NnSS2*. Data are shown as means ± SD (*n* = 3). **F)** Left, quantitative analysis. Right, dual-LUC activity assay shows that NnABI5 activates the expression of *NnSBEII*. Data are shown as means ± SD (*n* = 3). The experiments were performed independently twice with similar results, and one representative result is shown. Statistical analysis was performed using a one-way ANOVA, which is applicable to all statistical analyses in this figure. Significant levels are denoted by asterisks (two-sided Student's *t*-test; ****P < 0.0001).

**Figure 7.**
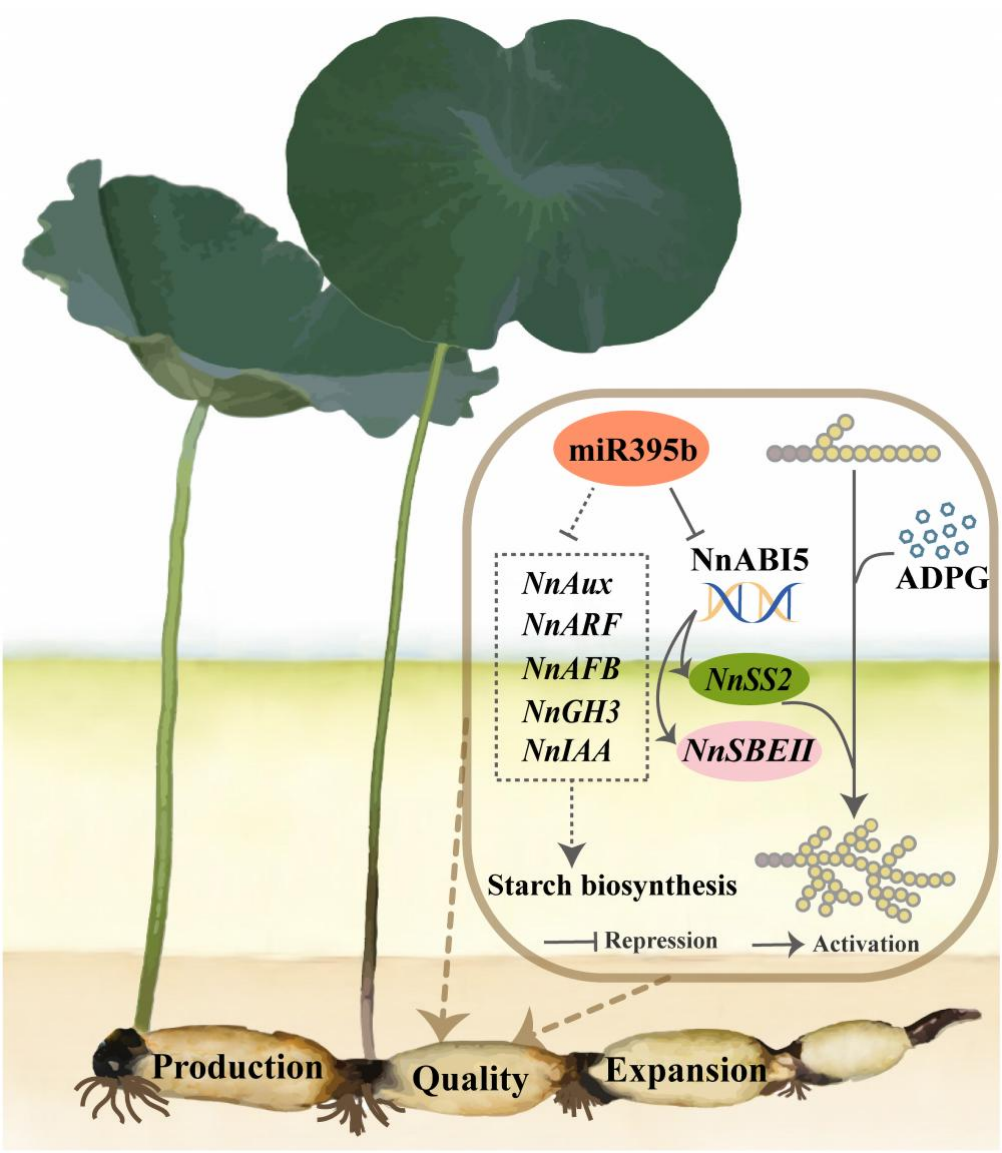
Model describing how the miR395b–NnABI5 pathway induces the starch biosynthesis in lotus roots. In this model, miR395b inhibits the transcription of NnABI5, while NnABI5 directly binds to and promotes the expression of *NnSS2* and *NnSBEII* promoters, affecting amylopectin accumulation and the distribution of amylopectin chain lengths, ultimately influencing the quality and yield of lotus roots. Solid lines represent direct regulation. Dashed lines represent multistep syntheses.

We performed yeast one-hybrid (Y1H) experiments to search for downstream target genes regulated by NnABI5. The full-length CDS of the *NnABI5* gene was inserted into the pGADT7 vector, and the *NnGBSSⅡa*, *NnSS2*, *NnSBEI*, and *NnSBEII* promoter regions were inserted into the pAbAi vector, and were respectively cotransfected with pGADT7-NnABI5 into yeast cells, and cultured in SD/–Leu+Aureobasidin A (AbA, 200 ng/mL) medium. pGADT7-NnABI5 grew normally in SD/–Leu+AbA (200 ng/mL) medium when cotransfected with pAbAi-*NnSS2* and pAbAi-*NnSBEII* respectively, but pGADT7 did not when cotransfected, indicating that NnABI5 binds to both *NnSS2* and *NnSBEII* promoter regions (Fig. 6, A and B). Therefore, we performed an electrophoretic mobility shift assay (EMSA) to ask whether NnABI5 can directly bind to the 5′-CACGTG-3′ motif in the *NnSS2* and *NnSBEII* promoters. Accordingly, we purified recombinant His-protein as negative control and His-NnABI5 from *Escherichia coli* cells and incubated both proteins with a biotin-labeled probe containing the His. We detected a shift in the labeled probes specifically with His-NnABI5 indicating the formation of a protein–DNA complex. The intensity of the shifted band was markedly weakened with the addition of increasing concentrations of unlabeled probe as competitor. By contrast, when an equal amount of a biotin-labeled probe harboring the A allele of the *NnSS2* and *NnSBEII* promoters were incubated with recombinant His-NnABI5, we did not observe the shifted band (Fig. 6, C and D). These results reveal that NnABI5 directly binds to the 5′-CACGTG-3′ motif of the *NnSS2* and *NnSBEII* promoters in vitro. To validate these results, we performed a dual-LUC assay in tobacco leaves. As reporter construct, we placed LUC under the control of the *NnSS2* and *NnSBEII* promoters. When we coinfiltrated the *NnSS2pro:LUC* and *NnSBEIIpro:LUC* reporters with the effector construct *NnABI5*-SK, we measured a significantly higher LUC/REN ratio than with the empty SK control vector (Fig. 6, E and F). In addition, we mutated the polymorphic nucleotide in the *NnSS2* and *NnSBEII* promoters from G (5′-CACGTG-3′) to A (5′-CACATA-3′) to verify the specific binding of NnABI5 to the 5′-CACGTG-3′ motif in the *NnSS2* and *NnSBEII* promoters (Supplementary Fig. S7, G and I). The detection showed that when *NnABI5*-SK was coinfiltrated with *NnSS2mpro:LUC* and *NnSBEIImpro:LUC* reporters, there was no significant difference in fluorescence intensity and LUC/REN ratio compared to the empty SK control vector, indicating that NnABI5 specifically binds to the ABRE motif (Supplementary Fig. S7, H and J). In summary, we demonstrate that NnABI5 acts as a transcriptional activator, regulates the biosynthesis and accumulation of amylopectin in lotus roots by binding *NnSS2* and *NnSBEII*, thereby influencing the quality of lotus roots. The decrease in miR395b levels lead to enhanced transcriptional activity of NnABI5, increased the accumulation of NnSS2 and NnSBEII enzymes, promoted amylopectin biosynthesis in lotus roots. This study reveals a key miRNA regulatory mechanism that can be further used to improve the yield and quality of lotus roots (Fig. 7).

## Discussion

Starch is the most crucial component in lotus roots, and its content directly impacts yield and quality. However, its physicochemical properties are the primary determinants of its consumption. miRNAs influence various metabolic pathways in plants, including seed germination, morphogenesis, flower and fruit development, and hormone signaling transduction (Samad et al. 2017; Yang et al. 2024). In this study, the expression of miR395b in “MRH,” which had a low starch content in lotus roots, was significantly higher than that in “EL-5,” which had a high starch content (Fig. 1A). When miR395b was transiently overexpressed, the amylopectin and total starch contents significantly decreased in lotus roots (Fig. 1, G and H). The proportion of short-chain amylopectin with a DP of 6 to 12 was increased, while the proportion of medium- and long-chain amylopectin with a DP of 12 to 24 reduced (Fig. 1E). After functional inhibiting miR395b, the starch content and the distribution ratio of amylopectin chain lengths in lotus roots showed the opposite trend compared with that observed when it was overexpressed. These results indicate that miR395b negatively regulates amylopectin synthesis and overall starch accumulation in lotus roots, and results in the formation of more short chains and fewer medium- and long-branched chains of amylopectin.

miRNAs play a significant role in modulating plant hormone signaling pathways and, thus, are closely associated with reproductive development. miR160 and miR167 regulate *ARF* genes expression, which influence growth, development, and adventitious root formation in *A. thaliana* (Liu et al. 2007; Gutierrez et al. 2009; Luo et al. 2022a). miR393 targets the *TIR* and *AFB* genes, which are auxin signaling pathway-related genes that influence root system development, plant height, tillering, grain starch content, and yield in rice (He et al. 2018; Guo et al. 2021; Wu et al. 2024). Auxin is closely related to carbohydrate allocation and starch synthesis. In rice, OsIAAs-OsARFs mediate auxin signaling cascade regulating carbohydrate partitioning and reproductive organ development between the source and sink tissues, and affect rice grain size and yield (Zhao et al. 2022b; Chen et al. 2024b; Xian et al. 2025). Auxin control sugar transport and unloading by regulating dorsal vascular bundle development, consequently affecting endosperm development in rice grains (Yu et al. 2024). In rice and wheat, ABA can increase grain filling rate, starch content, and dry matter accumulation, and prolong the filling period (Yang et al. 2006, 2014). ABA enhances the transcriptional activity of *MeSUS1*, *MeGBSSIa*, and *MeSBE2.4* by inducing high expression of *MeSnRK2.3* kinase, promoting the biosynthesis and accumulation of starch in cassava (Li et al. 2024). This study found that *MIR395b*-OE reduced the amylopectin and total starch contents of tobacco leaves, and the leaf length, width, and number of leaves were reduced, and the weight per plant was significantly lower than that of WT (Fig. 2). The present study found that miR395b increased the amylopectin and total starch contents and yield per plant of tobacco. *MIR395b*-OE tobacco transcriptome data showed that gene expression associated with auxin and ABA signaling pathway was significantly downregulated (Supplementary Table S2 and Supplementary Fig. S3). Based on previous studies, we speculate that miR395b responds to auxin and ABA signals by regulating the expression of genes associated with auxin and ABA signaling pathway, thereby influencing the starch synthesis, quality, and yield of lotus roots. Future research is required to substantiate this perspective.

miR395 is present in a variety of plants, including dicotyledons (*A. thaliana*, citrus, apple, cucumber, and mustard) (Liang and Yu 2010; Xu et al. 2010; Liu et al. 2022; Bhardwaj et al. 2024) and monocotyledons (rice) (Yang et al. 2022). miR395 targets genes encoding the ATP sulfurylase gene *APS* and sulfate transporter gene *SULTR*, influencing sulfate accumulation and its distribution in rice and *Arabidopsis* (Liang et al. 2010; Zhang et al. 2013; Yang et al. 2022). In addition to these conserved *APS* and *SULTR* targets, the expression of metal cadmium tolerance genes such as *BnPCS1*, *BnHO1*, and *Sultr1;1* was upregulated in the *35S:MIR395* kale-type oilseed rape strain and shown to be involved in cadmium detoxification in *Brassica napus* (Zhang et al. 2013). In this study, we found that NnABI5 is an atypical target of miR395b. Therefore, it is crucial to determine the biological functions of NnABI5 and the impact of miR395b on NnABI5 regulation in lotus roots. We demonstrated that miR395b targets NnABI5 and inhibits the accumulation of its transcript using 5′ RLM-RACE sequencing and dual-luciferase assays in tobacco (Fig. 3). Moreover, the starch content and amylopectin chain length distribution in *MIR395b*-OE lotus roots were almost identical to those in *NnABI5*-TRV, indicating that miR395b almost completely abolished NnABI5 activity in lotus roots (Figs. 1 and 4). Transient *NnABI5* overexpression promoted the accumulation of amylopectin and total starch in lotus roots, while miR395b targeted and inhibited the expression of *NnABI5*, exerting a negative regulatory effect on starch synthesis (Supplementary Fig. S7). Therefore, miR395b may provide a target, NnABI5, for starch biosynthesis in lotus roots. NnABI5 may be one of the target genes of miR395b in the regulation of starch synthesis pathway, and whether overexpressing *MIR395b* in *NnABI5*-TRV can also regulate the biosynthesis of total starch and amylopectin remains to be studied. Interestingly, our RNA-seq revealed that two other transcription factors, NnRdRP6 and NnPDR, were both downregulated in *MIR395b*-OE, and that NnRdRP6 and NnPDR may also play the same role as NnABI5 in starch synthesis, or regulate auxin and ABA biosynthesis-related pathways (Fig. 3). In the future research, we need more evidences to confirm this idea.

ABI transcription factor family plays a significant role in regulating starch metabolism and accumulation. By applying exogenous ABA to *Arabidopsis* and apple, the key AREB transcription factors, located downstream of the ABA signaling pathway, was activated, inducing the expression of amylase genes *BAM1*, *AMY1* and *AMY3*, and consequently accelerating starch degradation (Thalmann et al. 2016; Ma et al. 2017). In trifoliate orange, ABF4 and ABR1 interact to synergistically upregulate *BAM3* expression, thereby promoting starch degradation and sugar accumulation (Zhang et al. 2023). The research on the ABI5 transcription factor has mostly focused on the processes of seed maturation, dormancy, and germination within the ABA signaling pathway (Chen et al. 2020; Zhao et al. 2020). Additionally, light and abiotic stress antagonistically regulate the expression of ABI5 and its homologs, thereby affecting the plant's response to age-dependent senescence and dynamic environmental conditions (Sakuraba et al. 2014). Reports on the involvement of the ABI5 transcription factor in starch biosynthesis and its impact on yield and quality traits are still limited. In this study, we found that *NnABI5* overexpression promoted the accumulation of amylopectin and total starch in lotus roots, which is consistent with our previous findings on the effects of the *ABI* gene family member NnABI4 on starch accumulation in lotus roots (Wu et al. 2021). In addition, *NnABI5* overexpression significantly increased *NnSS2* and *NnSBEII* expression levels (Fig. 5). Subsequently, the Y1H system revealed that NnABI5 interacts with *NnSS2* and *NnSBEII* (Fig. 6, A and B). Further EMSA and tobacco dual-luciferase reporter assays showed that NnABI5 can directly bind to the ABRE sequences in *NnSS2* and *NnSBEⅡ* promoters and activate their expression (Fig. 6, C to F). Studies in wheat, barley (sex6), rice, and maize (sugary2) have shown that the *SS2* genes are responsible for the synthesis of long amylopectin chains and have similar functions in storage starch synthesis among crops (Yamamori and Endo 1996; Zhang et al. 2004; Irshad et al. 2021). SBE is responsible for catalyzing the formation of the α-1,6 glycosidic bond. In wheat kernel development, *SBE2* is responsible for catalyzing the formation of amylopectin branches in early stages, and *SBE1* is responsible for catalyzing starch synthesis in later stages (Nair et al. 1997; Wang and Sun 2023). A comprehensive analysis of our results and those studies in other species indicates that NnABI5 promotes amylopectin accumulation and the synthesis of medium- and long-chain amylopectin by increasing the transcriptional levels of *NnSS2* and *NnSBEⅡ*. This study explores and enriches the potential biological functions of lotus ABI5 family, providing more molecular regulatory mechanisms for the biosynthesis of plant starch.

## Conclusion

miR395b has a negative regulatory effect on amylopectin synthesis in lotus roots by targeting and cleaving NnABI5 transcripts, significantly reducing the expression of the amylopectin biosynthesis-related genes *NnSS2* and *NnSBEII*. However, miR395b potentially targets genes involved in auxin and ABA signaling transduction pathway, modulating starch biosynthesis and yield within lotus roots (Fig. 7). Our research findings not only provide molecular mechanisms for the quality improvement of lotus roots, but also reveal the genetic role of the ABI5 transcription factor family in regulating the development of plant organs development. In addition, further research is needed to explore the potential applications of miR395b in terms of lotus roots expansion and yield.

## Materials and methods

### Plant materials

Lotus resources “EL-5” and “MRH” were selected for the experiment, and planted in the experimental field of aquatic vegetables in the College of Horticulture and Landscape Architecture of Yangzhou University, under normal field management. *N. benthamiana* was grown in pots containing peat soil:vermiculite (1:1, v/v) under 24 ± 1 ℃, 50% relative humidity, 16 h of light/8 h of dark photoperiod, and 100 *μ*mol/m^2^/s LED lights.

### Virus-mediated gene silencing and transient expression

Virus-mediated gene silencing (VIGS) was performed as described in Zhang et al. (2019). A specific fragment of the miR395b-STTM (a short tandem target mimic of miR395b, 96-bp) containing *Sac*I and *Xba*I restriction sites at both ends was constructed into the pCAMBIA1300 vector. A specific fragment of the *NnABI5* (420-bp) gene containing *Eco*RI and *Xhol*I restriction sites at both ends was used to construct the plasmid *NnABI5*-pTRV2. *Agrobacterium tumefaciens* strain GV3101 carrying miR395b-STTM or *NnABI5*-pTRV2, respectively, are grown in Luria-Bertani (LB) medium containing 50 mg/L kanamycin and 25 mg/L rifampicin and shaken at 200 rpm for 16 h at 28 °C. *Agrobacterium* cells were collected by centrifugation at 5,000 rpm for 5 min at room temperature and resuspended in sterile infection solution (10 mm 2-(*N*-Morpholino) -ethanesulfonic acid, 10 mm MgCl_2_, 200 mm acetosyringone, pH 5.6), adjust the concentration to OD_600_ = 1.0 to 1.5, and incubate for 3 to 4 h at room temperature in the dark.

Lotus roots were selected at the initial swelling stage (with the first significantly enlarged lotus root segment, about 3 cm in diameter). In a laminar flow cabinet, the lotus roots were disinfected with 75% ethanol for 1 min and 3% sodium hypochlorite for 15 min, shaking every 2 to 3 min, and washed three times with sterile water for 5 min. The disinfected lotus roots were cut into 0.5-cm-thick slices and soaked in *A. tumefaciens* strain GV3101 infection solution containing the target gene. The slices were kept in the dark for 30 min, shaken every 10 min, and washed with sterile water. After drying, the slices were placed onto Murashige and Skoog (MS) solid medium and cultured in the dark for 3 d. At the end of the culture period, the transcriptional levels of miR395b or NnABI5 in the lotus roots were measured using RT-qPCR. The primers used in the STTM and VIGS assay are listed in Supplementary Table S3.

### RNA extraction and RT-qPCR

The miRNA was extracted from the lotus roots using the miRNA Extraction and Isolation Kit (TIANGEN, Beijing, China). cDNA was synthesized using the miR395b stem-loop primer and SuperScript Ⅲ RT-PCR (Vazyme, Nanjing, China), and the lotus U6 of lotus was used as the internal reference gene of miRNA, and the expression of mature miR395b was detected by using the miRNA-specific forward primer, generalized reverse primer, and stem-loop RT-qPCR.

Total RNA was extracted from lotus roots petals by a Isolation Kit (Vazyme, RC411-01) (Wang et al. 2020). Reverse transcription was performed with 1 *μ*g total RNA using HiScript II Q Select RT SuperMix kit (Vazyme, Nanjing, China). qPCR was performed on a CFX connect Real-Time System (Bio-Rad, Shanghai, China) and a ChamQ SYBR qPCR Master Mix was used to conduct qPCR experiments (Vazyme, Nanjing, China). *NnTBP* (LOC104611161) gene was used as an internal standard for mRNA gene expression normalization. All reactions were run with three independent biological replicates, and the relative gene expression levels were calculated using the 2^−ΔΔCt^ method (Livak and Schmittgen 2001). Primers used in RT-qPCR are listed in Supplementary Table S3.

### Analysis of starch characteristics and physicochemical properties

The instantaneously transformed lotus roots were stained with I_2_–KI to observe changes in starch content. The lotus roots were ground with liquid nitrogen, and the resulting powder was used for the determination of starch content. Total starch (No. BC0700) and amylopectin content (No. BC4270) using a starch content assay kit (Solaibao, Beijing, China). The chain length distribution of amylopectin was determined by Thermo ICS5000 Ion Chromatography System (ICS500+, Thermo Fisher Scientific, USA).

### 5′ RNA ligase-mediated rapid amplification of cDNA ends (5′ RLM-RACE)

The 5′ RLM-RACE assay was performed according to the manual of the FirstChoice RLM-RACE (Thermofisher, AM1700). Total RNA was treated with calf intestine alkaline phosphatase (CIP) and tobacco acid pyrophosphatase, and ligated with RNA adapters at 37 °C for 1 h. Reverse transcription and nested PCR for 5′ RLM-RACE (outer and inner) PCR were performed according to the manufacturer's instructions. The second PCR products were ligated into the TA Cloning Vector (5 min TA/Blunt-Zero Cloning Kit; Vazyme, Nanjing, China) for sequencing. Primers used in RLM-RACE are listed in Supplementary Table S3.

### Dual-luciferase reporter gene assay

To verify the transcriptional inhibiting NnABI5 by *MIR395b*, the *MIR395bpre*-SK effector plasmid was obtained by inserting the precursor sequence of *MIR395b* into the pGreenII 62-SK vector, and the sequence that could be matched with *MIR395b* in the *NnABI5* sequence was extended by 200-bp before and after to the pGreenII 0800-miRNA vector to obtain the *LUC*-*NnABI5* reporter plasmid. We mutated the polymorphic nucleotide in the *NnABI5* sequence from 5′-GAATTTCTCGAAACATTTCGG-3′ to 5′-GCAAAATAGGAACCACTTGGG-3′ to obtain the *LUC*-*NnABI5m* reporter plasmid.

To verify the transcriptional regulation of NnABI5 on *NnSS2* and *NnSBEII*, the CDS sequence of *NnABI5* was inserted into the pGreenII 62-SK vector to obtain the *NnABI5*-SK effector plasmid, and the promoter sequences of *NnSS2* and *NnSBEII* were constructed into the pGreenII 0800-LUC vector to obtain the *NnSS2pro:LUC* and *NnSBEIIpro:LUC* reporter plasmids. In addition, we mutated the polymorphic nucleotide in the *NnSS2* and *NnSBEII* promoters from G (5′-CACGTG-3′) to A (5′-CACATA-3′) to obtain the *NnSS2mpro:LUC* and *NnSBEIImpro:LUC* reporter plasmids.

The above recombinant plasmids were transformed into *Agrobacterium* strain GV3101 and mixed at a ratio of 1:1 to jointly infect tobacco leaves. After 3 d of infiltration, luciferase activity was analyzed using a luminescence detector (Promega Glomax 2020, Promega, USA). The ratio between firefly (LUC) and *Renilla* (REN) luciferase activity is measured using dual-LUC detection reagents (Promega, Madison, WI, USA). Primers for LUC analysis are listed in Supplementary Table S3.

### Y2H assay

The CDS sequence of *NnABI5* was cloned into the pGBKT7 vector, and the resulting pGBKT7-NnABI5 recombinant plasmid was transformed into the Matchmaker Gold Y2H Library Screening System (Clontech, Dalian, China) for Y2H assays. The growth status of the yeast was then assessed using synthetic defined (SD) medium lacking Trp and His (SD/–Trp–His) or Trp and Ade (SD/–Trp–Ade). Primers for Y2H assay are listed in Supplementary Table S3.

### Y1H assay

The promoter fragments of *NnSS2* and *NnSBEⅡ* containing ABRE elements were ligated into the pAbAi vector. The full-length CDS sequence of *NnABI5* was cloned and inserted into the pGADT7 vector. The interaction between NnABI5 and the promoters of *NnSS2* and *NnSBEⅡ* was examined using the Matchmaker Gold Y1H Library Screening System (Clontech, Dalian, China) for Y1H assays. The constructed vectors were transformed into the yeast strain Y1H gold, which was subsequently cultured on SD medium lacking Ura and Leu (SD/–Ura–Leu), with or without the addition of 200 ng/mL AbA. Primers used in Y1H assay are listed in Supplementary Table S3.

### EMSA

The NnABI5 CDS was inserted into the pCold TF vector to generate His-NnABI5. The resulting vector was transformed into *E. coli* strain BL21 (DE3) to produce recombinant His-NnABI5 fusion protein, which was induced by the addition of isopropyl-β-D-thiogalactopyranoside (IPTG, 0.5 mm) at 16 °C for 16 h. The His-NnABI5 protein was purified with Ni-Agarose resin (Cowin Biotech Co., Ltd, Jiangsu, China) following the manufacturer's instructions. EMSAs were performed using a chemiluminescent nucleic acid detection module kit (Beyotime Biotechnology, Shanghai, China). The primers used in EMSA are listed in Supplementary Table S3.

### Accession numbers

The raw sequence data for RNA-seq was deposited in this article can be found in the China National GeneBank DataBase data libraries (https://db.cngb.org/) under accession number CNP0008177. Sequence data from this article can be found in the GenBank/EMBL data libraries (https://www.ncbi.nlm.nih.gov/). ABI5 (XM_010273373.2), RdRP6 (NW_010729080.1), PDR3 (NW_010729139.1), SS2 (NW_010729075.1), SBEII (NW_010729078.1).

## Supplementary Material

kiaf554_Supplementary_Data

## Data Availability

The data used and/or analyzed in this study are available in the manuscript and the accompanying supplementary data.

## Supplementary data

The following materials are available in the online version of this article.

**Supplementary Figure S1.** Structural analysis of miR395 family precursors and miR395b silencing in lotus roots.

**Supplementary Figure S2.** PCR detection of transgenic plants and RNA transcriptome analysis of WT and *MIR395b*-OE tobacco lines.

**Supplementary Figure S3.** Cluster heatmap analysis of differentially expressed genes between WT and *MIR395b*-OE tobacco lines.

**Supplementary Figure S4.** RNA transcriptome analysis of “EL-5” and “MRH.”

**Supplementary Figure S5.** Characterization of NnABI5.

**Supplementary Figure S6.** Bioinformatics analysis of ABI5.

**Supplementary Figure S7.** NnABI5 positively regulates starch biosynthesis in lotus roots and is inhibited by miR395b.

**Supplementary Table S1.** The sequences and minimum free energy (MFE) of miR395 precursors were predicted.

**Supplementary Table S2.** Genes associated with auxin and ABA signaling in the tobacco transcriptome.

**Supplementary Table S3.** List of primers used.

## Dive Curated Terms

The following phenotypic, genotypic, and functional terms are of significance to the work described in this paper:

ABF4 Gramene: AT3G19290
ABF4 Araport: AT3G19290

ABR1 Gramene: AT5G64750
ABR1 Araport: AT5G64750

MIR395b Gramene: AT1G26975
MIR395b Araport: AT1G26975

AMY1 Gramene: AT4G25000
AMY1 Araport: AT4G25000

AtSS2 Gramene: AT3G01180
AtSS2 Araport: AT3G01180

BAM3 Gramene: AT4G20270
BAM3 Araport: AT4G20270

SHB1 Gramene: AT4G25350
SHB1 Araport: AT4G25350

SS2 Gramene: AT1G74020
SS2 Araport: AT1G74020

SULTR1;1 Gramene: AT4G08620
SULTR1;1 Araport: AT4G08620

AMY3 Gramene: AT1G69830
AMY3 Araport: AT1G69830

BAM1 Gramene: AT3G23920
BAM1 Araport: AT3G23920

## References

[kiaf554-B1] Bhardwaj E, Pokhriyal E, Jain A, Lal M, Khari M, Jalan K, Das S. The non-canonically organized members of *MIR395* gene family in *Brassica juncea* are associated with developmentally regulated, sulfate-stress responsive bidirectional promoters that exhibit orientation-dependent differential transcriptional activity. Plant Sci. 2024:348:112214. 10.1016/j.plantsci.2024.112214 39127349

[kiaf554-B2] Bi C, Ma Y, Wu Z, Yu Y, Liang S, Lu K, Wang XF. *Arabidopsis* ABI5 plays a role in regulating ROS homeostasis by activating *CATALASE 1* transcription in seed germination. Plant Mol Biol. 2017:94(1-2):197–213. 10.1007/s11103-017-0603-y 28391398 PMC5437177

[kiaf554-B3] Carles C, Bies-Etheve N, Aspart L, Léon-Kloosterziel KM, Koornneef M, Echeverria M, Delseny M. Regulation of *Arabidopsis thaliana Em* genes: role of ABI5. Plant J. 2002:30(3):373–383. 10.1046/j.1365-313X.2002.01295.x 12000684

[kiaf554-B4] Chen K, Li GJ, Bressan RA, Song CP, Zhu JK, Zhao Y. Abscisic acid dynamics, signaling, and functions in plants. J Integr Plant Biol. 2020:62(1):25–54. 10.1111/jipb.12899 31850654

[kiaf554-B5] Chen X, Han CZ, Yang RR, Wang XW, Ma JZ, Wang YG. Influence of the transcription factor ABI5 on growth and development in *Arabidopsis*. J Plant Physiol. 2024a:302:154316. 10.1016/j.jplph.2024.154316 39098091

[kiaf554-B6] Chen ZH, Zhou W, Guo XY, Ling S, Li W, Wang X, Yao JL. Heat stress responsive Aux/IAA protein, OsIAA29 regulates grain filling through OsARF17 mediated auxin signaling pathway. Rice (N Y). 2024b:17(1):16. 10.1186/s12284-024-00694-z 38374238 PMC10876508

[kiaf554-B7] Cheng ZJ, Zhao XY, Shao XX, Wang F, Zhou C, Liu YG, Zhang Y, Zhang XS. Abscisic acid regulates early seed development in *Arabidopsis* by ABI5-mediated transcription of *SHORT HYPOCOTYL UNDER BLUE1*. Plant Cell. 2014:26(3):1053–1068. 10.1105/tpc.113.121566 24619610 PMC4001368

[kiaf554-B8] Copeland L, Blazek J, Salman H, Tang MC. Form and functionality of starch. Food Hydrocoll. 2009:23(6):1527–1534. 10.1016/j.foodhyd.2008.09.016

[kiaf554-B9] Feng MQ, Lu MD, Long JM, Yin ZP, Jiang N, Wang PB, Liu Y, Guo WW, Wu XM. Mir156 regulates somatic embryogenesis by modulating starch accumulation in citrus. J Exp Bot. 2022:73(18):6170–6185. 10.1093/jxb/erac248 35661206

[kiaf554-B10] Goldstein A, Annor G, Putaux JL, Hebelstrup KH, Blennow A, Bertoft E. Impact of full range of amylose contents on the architecture of starch granules. Int J Biol Macromol. 2016:89:305–318. 10.1016/j.ijbiomac.2016.04.053 27109754

[kiaf554-B11] Guo F, Huang YZ, Qi PP, Lian GW, Hu XM, Han N, Wang JH, Zhu MY, Qian Q, Bian HW. Functional analysis of auxin receptor *OsTIR1*/*OsAFB* family members in rice grain yield, tillering, plant height, root system, germination, and auxinic herbicide resistance. New Phytol. 2021:229(5):2676–2692. 10.1111/nph.17061 33135782

[kiaf554-B12] Gutierrez L, Bussell JD, Pacurar DI, Schwambach J, Pacurar M, Bellini C. Phenotypic plasticity of adventitious rooting in *Arabidopsis* is controlled by complex regulation of AUXIN RESPONSE FACTOR transcripts and microRNA abundance. Plant Cell. 2009:21(10):3119–3132. 10.1105/tpc.108.064758 19820192 PMC2782293

[kiaf554-B13] He Q, Yang L, Hu W, Zhang J, Xing YZ. Overexpression of an auxin receptor *OsAFB6* significantly enhanced grain yield by increasing cytokinin and decreasing auxin concentrations in rice panicle. Sci Rep. 2018:8(1):14051. 10.1038/s41598-018-32450-x 30232356 PMC6145926

[kiaf554-B14] He ST, Hao XM, Wang SS, Zhou WZ, Ma QX, Lu XL, Chen LN, Zhang P. Starch synthase II plays a crucial role in starch biosynthesis and the formation of multienzyme complexes in cassava storage roots. J Exp Bot. 2022:73(8):2540–2557. 10.1093/jxb/erac022 35134892

[kiaf554-B15] Hu YF, Li YP, Weng JF, Liu HM, Yu GW, Liu YH, Xiao QL, Huang HH, Wang YB, Wei B, et al Coordinated regulation of starch synthesis in maize endosperm by microRNAs and DNA methylation. Plant J. 2021:105(1):108–123. 10.1111/tpj.15043 33098697

[kiaf554-B16] Huang LC, Tan HY, Zhang CQ, Li QF, Liu QQ. Starch biosynthesis in cereal endosperms: an updated review over the last decade. Plant Commun. 2021:2(5):100237. 10.1016/j.xplc.2021.100237 34746765 PMC8554040

[kiaf554-B17] Ibarra SE, Tognacca RS, Dave A, Graham IA, Sánchez RA, Botto JF. Molecular mechanisms underlying the entrance in secondary dormancy of *Arabidopsis* seeds. Plant Cell Environ. 2016:39(1):213–221. 10.1111/pce.12607 26177669

[kiaf554-B18] Irshad A, Guo HJ, Rehman SU, Wang XQ, Wang CJ, Raza A, Zhou CY, Li YT, Liu LX. Soluble starch synthase enzymes in cereals: an updated review. Agronomy. 2021:11(10):100237. 10.3390/agronomy11101983

[kiaf554-B19] Jones-Rhoades MW, Bartel DP, Bartel B. MicroRNAS and their regulatory roles in plants. Annu Rev Plant Biol. 2006:57(1):19–53. 10.1146/annurev.arplant.57.032905.105218 16669754

[kiaf554-B20] Li K, Li YJ, Liu C, Li MT, Bao RX, Wang HY, Zeng CY, Zhou XC, Chen YH, Wang WQ, et al Protein kinase MeSnRK2.3 positively regulates starch biosynthesis by interacting with the transcription factor MebHLH68 in cassava. J Exp Bot. 2024:75(20):6369–6387. 10.1093/jxb/erae343 39139055

[kiaf554-B21] Li LH, Yi HL, Xue MZ, Yi M. Mir398 and miR395 are involved in response to SO_2_ stress in *Arabidopsis thaliana*. Ecotoxicology. 2017:26(9):1181–1187. 10.1007/s10646-017-1843-y 28819808

[kiaf554-B22] Liang G, Yang FX, Yu DQ. MicroRNA395 mediates regulation of sulfate accumulation and allocation in *Arabidopsis thaliana*. Plant J. 2010:62(6):1046–1057. 10.1111/j.1365-313X.2010.04216.x 20374528

[kiaf554-B23] Liang G, Yu DQ. Reciprocal regulation among miR395, *APS* and *SULTR2;1* in *Arabidopsis thaliana*. Plant Signal Behav. 2010:5(10):1257–1259. 10.4161/psb.5.10.12608 20935495 PMC3115361

[kiaf554-B24] Liu CH, Ma D, Wang ZH, Chen NC, Ma XY, He XQ. Mir395c regulates secondary xylem development through sulfate metabolism in poplar. Front Plant Sci. 2022:13:897376. 10.3389/fpls.2022.897376 35755696 PMC9218717

[kiaf554-B25] Liu PP, Montgomery TA, Fahlgren N, Kasschau KD, Nonogaki H, Carrington JC. Repression of *AUXIN RESPONSE FACTOR10* by microRNA160 is critical for seed germination and post-germination stages. Plant J. 2007:52(1):133–146. 10.1111/j.1365-313X.2007.03218.x 17672844

[kiaf554-B26] Livak KJ, Schmittgen TD. Analysis of relative gene expression data using real-time quantitative PCR and the 2^−ΔΔCT^ method. Methods. 2001:25(4):402–408. 10.1006/meth.2001.1262 11846609

[kiaf554-B27] Lunn JE, MacRae E. New complexities in the synthesis of sucrose. Curr Opin Plant Biol. 2003:6(3):208–214. 10.1016/S1369-5266(03)00033-5 12753969

[kiaf554-B28] Luo P, Di DW, Wu L, Yang JW, Lu YF, Shi WM. MicroRNAs are involved in regulating plant development and stress response through fine-tuning of TIR1/AFB-dependent auxin signaling. Int J Mol Sci. 2022a:23(1):510. 10.3390/ijms23010510 35008937 PMC8745101

[kiaf554-B29] Luo S, Ma QX, Zhong YY, Jing JL, Wei ZS, Zhou WZ, Lu XL, Tian YN, Zhang P. Editing of the starch branching enzyme gene *SBE2* generates high-amylose storage roots in cassava. Plant Mol Biol. 2022b:108(4-5):429–442. 10.1007/s11103-021-01215-y 34792751

[kiaf554-B30] Ma QJ, Sun MH, Lu J, Liu YJ, Hu DG, Hao YJ. Transcription factor AREB2 is involved in soluble sugar accumulation by activating sugar transporter and amylase genes. Plant Physiol. 2017:174(4):2348–2362. 10.1104/pp.17.00502 28600345 PMC5543958

[kiaf554-B31] Meng FR, Liu H, Wang KT, Liu L, Wang SH, Zhao YH, Yin J, Li YC. Development-associated microRNAs in grains of wheat (*Triticum aestivum* L.). BMC Plant Biol. 2013:13(1):140. 10.1186/1471-2229-13-140 24060047 PMC4015866

[kiaf554-B32] Miura S, Koyama N, Crofts N, Hosaka Y, Abe M, Fujita N. Generation and starch characterization of non-transgenic BEI and BEIIb double mutant rice (*Oryza sativa*) with ultra-high level of resistant starch. Rice. 2021:14(1):3. 10.1186/s12284-020-00441-0 33409744 PMC7788159

[kiaf554-B33] Morita R, Crofts N, Miura S, Ikeda KI, Aoki N, Fukayama H, Fujita N. Characterization of the functions of starch synthase IIIb expressed in the vegetative organs of rice (*Oryza sativa* L.). Plant Cell Physiol. 2023:64(1):94–106. 10.1093/pcp/pcac143 36222360

[kiaf554-B34] Naguleswaran S, Vasanthan T, Hoover R, Bressler D. Amylolysis of amylopectin and amylose isolated from wheat, triticale, corn and barley starches. Food Hydrocoll. 2014:35:686–693. 10.1016/j.foodhyd.2013.08.018

[kiaf554-B35] Nair RB, Båga M, Scoles GJ, Kartha KK, Chibbar RN. Isolation, characterization and expression analysis of a starch branching enzyme II cDNA from wheat. Plant Sci. 1997:122(2):153–163. 10.1016/S0168-9452(96)04543-8

[kiaf554-B36] Patterson JA, Tetlow IJ, Emes MJ. Bioinformatic and in vitro analyses of arabidopsis starch synthase 2 reveal post-translational regulatory mechanisms. Front Plant Sci. 2018:9:1338. 10.3389/fpls.2018.01338 30283470 PMC6156364

[kiaf554-B37] Regina A, Kosar-Hashemi B, Ling S, Li ZY, Rahman S, Morell M. Control of starch branching in barley defined through differential RNAi suppression of starch branching enzyme IIa and IIb. J Exp Bot. 2010:61(5):1469–1482. 10.1093/jxb/erq011 20156842 PMC2837261

[kiaf554-B38] Rogers K, Chen XM. Biogenesis, turnover, and mode of action of plant microRNAs. Plant Cell. 2013:25(7):2383–2399. 10.1105/tpc.113.113159 23881412 PMC3753372

[kiaf554-B39] Sakuraba Y, Jeong J, Kang MY, Kim J, Paek NC, Choi G. Phytochrome-interacting transcription factors PIF4 and PIF5 induce leaf senescence in Arabidopsis. Nat Commun. 2014:5(1):4636. 10.1038/ncomms5636 25119965

[kiaf554-B40] Samad AFA, Sajad M, Nazaruddin N, Fauzi IA, Murad AMA, Zainal Z, Ismail I. MicroRNA and transcription factor: key players in plant regulatory network. Front Plant Sci. 2017:8:565. 10.3389/fpls.2017.00565 28446918 PMC5388764

[kiaf554-B41] Seung D, Smith AM. Starch granule initiation and morphogenesis-progress in Arabidopsis and cereals. J Exp Bot. 2019:70(3):771–784. 10.1093/jxb/ery412 30452691

[kiaf554-B42] Skubacz A, Daszkowska-Golec A, Szarejko I. The role and regulation of ABI5 (ABA-insensitive 5) in plant development, abiotic stress responses and phytohormone crosstalk. Front Plant Sci. 2016:7:1884. 10.3389/fpls.2016.01884 28018412 PMC5159420

[kiaf554-B43] Song ZY, Lai XH, Yao YL, Qin JJ, Ding XC, Zheng QL, Pang XQ, Chen WX, Li XP, Zhu XY. F-box protein EBF1 and transcription factor ABI5-like regulate banana fruit chilling-induced ripening disorder. Plant Physiol. 2022:188(2):1312–1334. 10.1093/plphys/kiab532 34791491 PMC8825429

[kiaf554-B44] Thalmann M, Pazmino D, Seung D, Horrer D, Nigro A, Meier T, Kölling K, Pfeifhofer HW, Zeeman SC, Santelia D. Regulation of leaf starch degradation by abscisic acid is important for osmotic stress tolerance in plants. Plant Cell. 2016:28(8):1860–1878. 10.1105/tpc.16.00143 27436713 PMC5006701

[kiaf554-B45] Tuncel A, Corbin KR, Ahn-Jarvis J, Harris S, Hawkins E, Smedley MA, Harwood W, Warren FJ, Patron NJ, Smith AM. Cas9-mediated mutagenesis of potato starch-branching enzymes generates a range of tuber starch phenotypes. Plant Biotechnol J. 2019:17(12):2259–2271. 10.1111/pbi.13137 31033104 PMC6835119

[kiaf554-B46] Wang L, Yang T, Lin QL, Wang BQ, Li X, Luan S, Yu F. Receptor kinase FERONIA regulates flowering time in *Arabidopsis*. BMC Plant Biol. 2020:20(1):26. 10.1186/s12870-019-2223-y 31948398 PMC6966814

[kiaf554-B47] Wang YL, Sun GL. Molecular prospective on the wheat grain development. Crit Rev Biotechnol. 2023:43(1):38–49. 10.1080/07388551.2021.2001784 34965821

[kiaf554-B48] Wu DX, Cao YN, Wang DJ, Zong GX, Han KX, Zhang W, Qi YH, Xu GH, Zhang YL. Auxin receptor OsTIR1 mediates auxin signaling during seed filling in rice. Plant Physiol. 2024:194(4):2434–2448. 10.1093/plphys/kiae013 38214208

[kiaf554-B49] Wu P, Liu AL, Zhang Y, Feng K, Zhao SP, Li LJ. NnABI4-mediated ABA regulation of starch biosynthesis in lotus (*Nelumbo nucifera* Gaertn). Int J Mol Sci. 2021:22(24):13506. 10.3390/ijms222413506 34948298 PMC8705639

[kiaf554-B50] Xian FJ, Liu SY, Huang JH, Xie B, Zhu L, Zhang QN, Lv C, Xu YM, Zhang XR, Hu J. The OsIAA3-OsARF16-*OsBUL1* auxin signaling module regulates grain size in rice. Plant Physiol. 2025:197(4):kiaf122. 10.1093/plphys/kiaf122 40156155

[kiaf554-B51] Xu Q, Liu YL, Zhu AD, Wu XM, Ye JL, Yu KQ, Guo WW, Deng XX. Discovery and comparative profiling of microRNAs in a sweet orange red-flesh mutant and its wild type. BMC Genomics. 2010:11(1):246. 10.1186/1471-2164-11-246 20398412 PMC2864249

[kiaf554-B52] Yamamori M, Endo TR. Variation of starch granule proteins and chromosome mapping of their coding genes in common wheat. Theor Appl Genet. 1996:93(1-2):275–281. 10.1007/BF00225757 24162229

[kiaf554-B53] Yang C, Li XB, Chen SQ, Liu CL, Yang LM, Li KL, Liao J, Zheng XN, Li HB, Li YQ, et al ABI5-FLZ13 module transcriptionally represses growth-related genes to delay seed germination in response to ABA. Plant Commun. 2023:4(6):100636. 10.1016/j.xplc.2023.100636 37301981 PMC10721476

[kiaf554-B54] Yang DQ, Luo YL, Ni YL, Yin YP, Yang WB, Peng DL, Cui ZY, Wang ZL. Effects of exogenous ABA application on post-anthesis dry matter redistribution and grain starch accumulation of winter wheat with different staygreen characteristics. Crop J. 2014:2(2-3):144–153. 10.1016/j.cj.2014.02.004

[kiaf554-B55] Yang JC, Zhang JH, Wang ZQ, Liu K, Wang P. Post-anthesis development of inferior and superior spikelets in rice in relation to abscisic acid and ethylene. J Exp Bot. 2006:57(1):149–160. 10.1093/jxb/erj018 16330527

[kiaf554-B56] Yang YZ, Xu L, Hao C, Wan MM, Tao YH, Zhuang Y, Su YN, Li L. The microRNA408- plantacyanin module balances plant growth and drought resistance by regulating reactive oxygen species homeostasis in guard cells. Plant Cell. 2024:36(10):4338–4355. 10.1093/plcell/koae144 38723161 PMC11448907

[kiaf554-B57] Yang ZY, Hui SG, Lv Y, Zhang MJ, Chen D, Tian JJ, Zhang HT, Liu HB, Cao JB, Xie WY, et al miR395-regulated sulfate metabolism exploits pathogen sensitivity to sulfate to boost immunity in rice. Mol Plant. 2022:15(4):671–688. 10.1016/j.molp.2021.12.013 34968734

[kiaf554-B58] Yu FF, Wu YR, Xie Q. Precise protein post-translational modifications modulate ABI5 activity. Trends Plant Sci. 2015:20(9):569–575. 10.1016/j.tplants.2015.05.004 26044742

[kiaf554-B59] Yu YC, Xu XM, Hu YX, Ding YF, Chen L. Indole-3-acetic acid (IAA) and sugar mediate endosperm development in rice (*Oryza sativa* L.). Rice (N Y). 2024:17(1):66. 10.1186/s12284-024-00745-5 39443408 PMC11499519

[kiaf554-B60] Zhang L, Zhao Y, Hu W, Qian JY, Ding XL, Guan CR, Lu YQ, Cao Y. Multi-scale structures of cassava and potato starch fractions varying in granule size. Carbohydr Polym. 2018:200:400–407. 10.1016/j.carbpol.2018.08.022 30177180

[kiaf554-B61] Zhang LW, Song JB, Shu XX, Zhang Y, Yang ZM. Mir395 is involved in detoxification of cadmium in *Brassica napus*. J Hazard Mater. 2013:250-251:204–211. 10.1016/j.jhazmat.2013.01.053 23454459

[kiaf554-B62] Zhang QL, Li Y, Zhang Y, Wu CB, Wang SN, Hao L, Wang SY, Li TZ. Md-miR156ab and md-miR395 target WRKY transcription factors to influence apple resistance to leaf spot disease. Front Plant Sci. 2017:8:526. 10.3389/fpls.2017.00526 28469624 PMC5395612

[kiaf554-B63] Zhang S, Feng M, Chen W, Zhou X, Lu J, Wang Y, Li Y, Jiang CZ, Gan SS, Ma N, et al In rose, transcription factor PTM balances growth and drought survival via PIP2;1 aquaporin. Nat Plants. 2019:5(3):290–299. 10.1038/s41477-019-0376-1 30833710

[kiaf554-B64] Zhang XL, Colleoni C, Ratushna V, Sirghie-Colleoni M, James MG, Myers AM. Molecular characterization demonstrates that the *Zea mays* gene *sugary2* codes for the starch synthase isoform SSIIa. Plant Mol Biol. 2004:54(6):865–879. 10.1007/s11103-004-0312-1 15604657

[kiaf554-B65] Zhang Y, Zhu J, Khan M, Wang Y, Xiao W, Fang T, Qu J, Xiao P, Li CL, Liu JH. Transcription factors ABF4 and ABR1 synergistically regulate amylase-mediated starch catabolism in drought tolerance. Plant Physiol. 2023:191(1):591–609. 10.1093/plphys/kiac428 36102815 PMC9806598

[kiaf554-B66] Zhao H, Nie K, Zhou H, Yan X, Zhan Q, Zheng Y, Song CP. ABI5 modulates seed germination via feedback regulation of the expression of the *PYR*/*PYL*/*RCAR* ABA receptor genes. New Phytol. 2020:228(2):596–608. 10.1111/nph.16713 32473058

[kiaf554-B67] Zhao SP, Deng KM, Zhu YM, Jiang T, Wu P, Feng K, Li LJ. Optimization of slow-release fertilizer application improves lotus rhizome quality by affecting the physicochemical properties of starch. J Integr Agric. 2023:22(4):1045–1057. 10.1016/j.jia.2023.01.005

[kiaf554-B68] Zhao SP, Ruan FJ, Shen WJ, Deng KM, Jiang T, Wu P, Feng K, Li LJ. The effect of nitrogen fertilizer on rhizome quality and starch physicochemical properties in *Nelumbo nucifera*. Agronomy. 2022a:12(4):794. 10.3390/agronomy12040794

[kiaf554-B69] Zhao SP, Zhang CY, Jiao J, Zhang Y, Jiang T, Wu P, Feng K, Li LJ. The transcription factor NnNAC100 positively regulates amylopectin biosynthesis by activating *NnSBEII* in the rhizome of *Nelumbo nucifera* Gaertn. Plant Cell Rep. 2025:44(1):21. 10.1007/s00299-024-03408-3 39751893

[kiaf554-B70] Zhao ZG, Wang CL, Yu XW, Tian YL, Wang WX, Zhang YH, Bai WT, Yang N, Zhang T, Zheng H, et al Auxin regulates source-sink carbohydrate partitioning and reproductive organ development in rice. Proc Natl Acad Sci U S A. 2022b:119(36):e2121671119. 10.1073/pnas.2121671119 36037381 PMC9457257

[kiaf554-B71] Zhu YM, Zhao SP, Deng K, Wu PM, Feng K, Li LJ. Integrated mRNA and small RNA sequencing reveals a microRNA regulatory network associated with starch biosynthesis in lotus (*Nelumbo nucifera* Gaertn.) rhizomes. Int J Mol Sci. 2022:23(14):7605. 10.3390/ijms23147605 35886954 PMC9318480

