# Supplementary material for: The miR395b–ABI5 module regulates amylopectin branching and biosynthesis and affects lotus root quality
Source: Plant Physiol. 2025 Nov 5;199(3):kiaf554. doi: 10.1093/plphys/kiaf554 (PMC12610401; doi:10.1093/plphys/kiaf554)
Supplement: kiaf554_Supplementary_Data [file kiaf554_supplementary_data.zip › Supplementary Data.pdf]

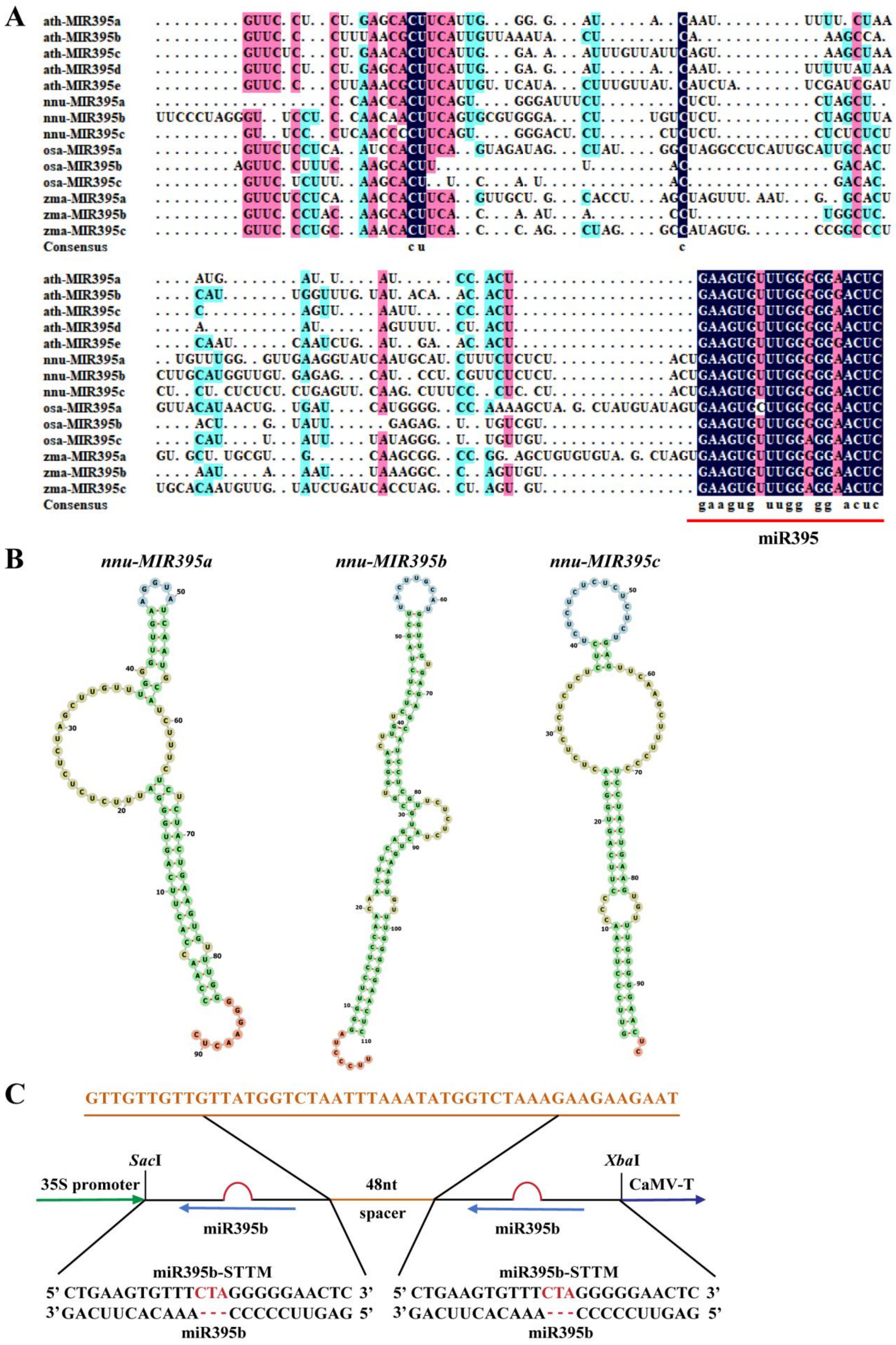

Supplementary Figure S1. Structural analysis of miR395 family precursors and miR395b silencing in lotus roots. **A)** Alignment analysis of the miR395 precursor sequences. Red line indicates the sequence of mature miR395. The bases with blue, red, and green backgrounds

respectively represents 100%, 80%, and 50% of identity. **B)** Secondary structure of the miR395 precursor sequence. **C)** Schematic representation of the short tandem target mimic (STTM) structure of miR395b inserted into the pCAMBIA1300 vector.

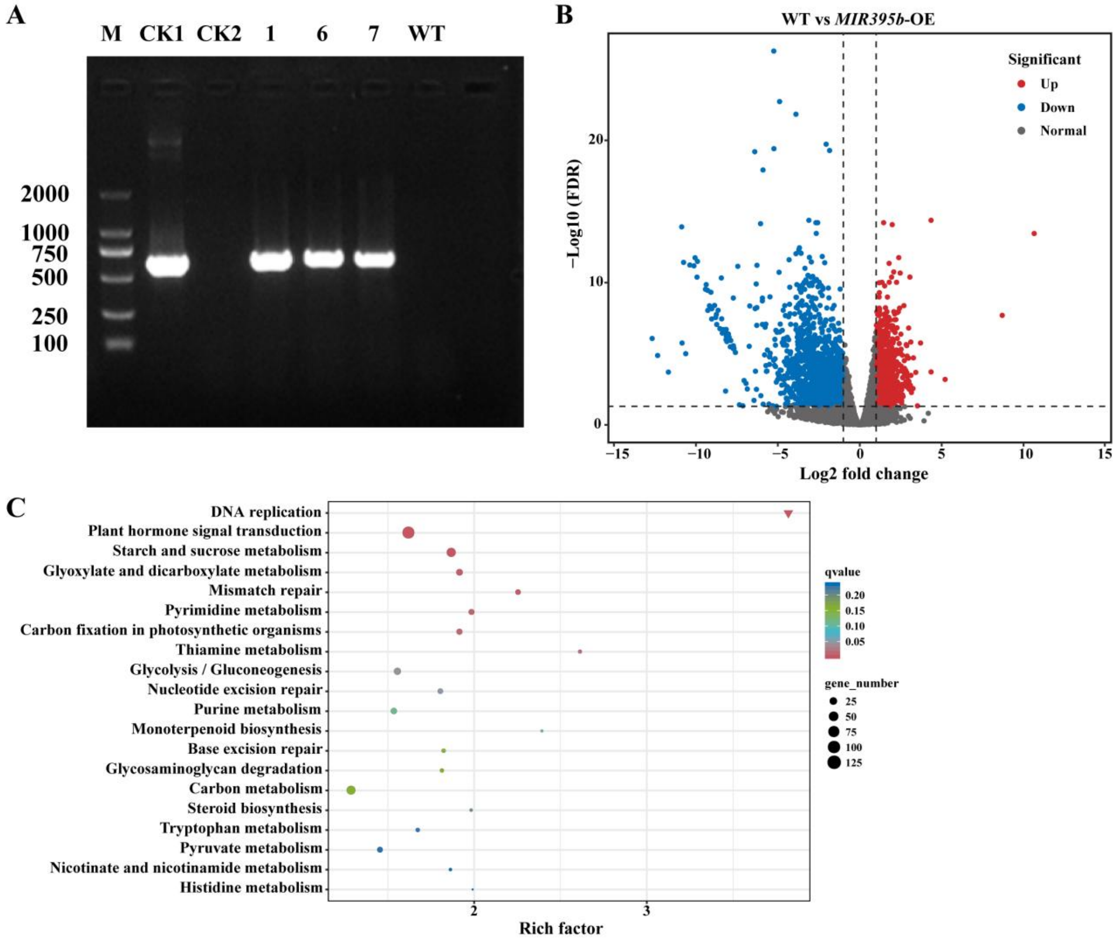

Supplementary Figure S2. PCR detection of transgenic plants and RNA transcriptome analysis of wild type (WT) and *MIR395b*-OE tobacco lines. OE: overexpression. **A)** PCR verification positive transgenic plants electrophoresis image. M: Marker DL2000; CK1: Positive control; CK2: Negative control; 1, 6, 7: Transformation plants. **B)** Number of down- and upregulated DEGs between WT and *MIR395b*-OE tobacco strains. **C)** Significantly enriched KEGG pathways in WT and *MIR395b*-OE. There were three biological replicates (n = 3) for RNA sequencing.

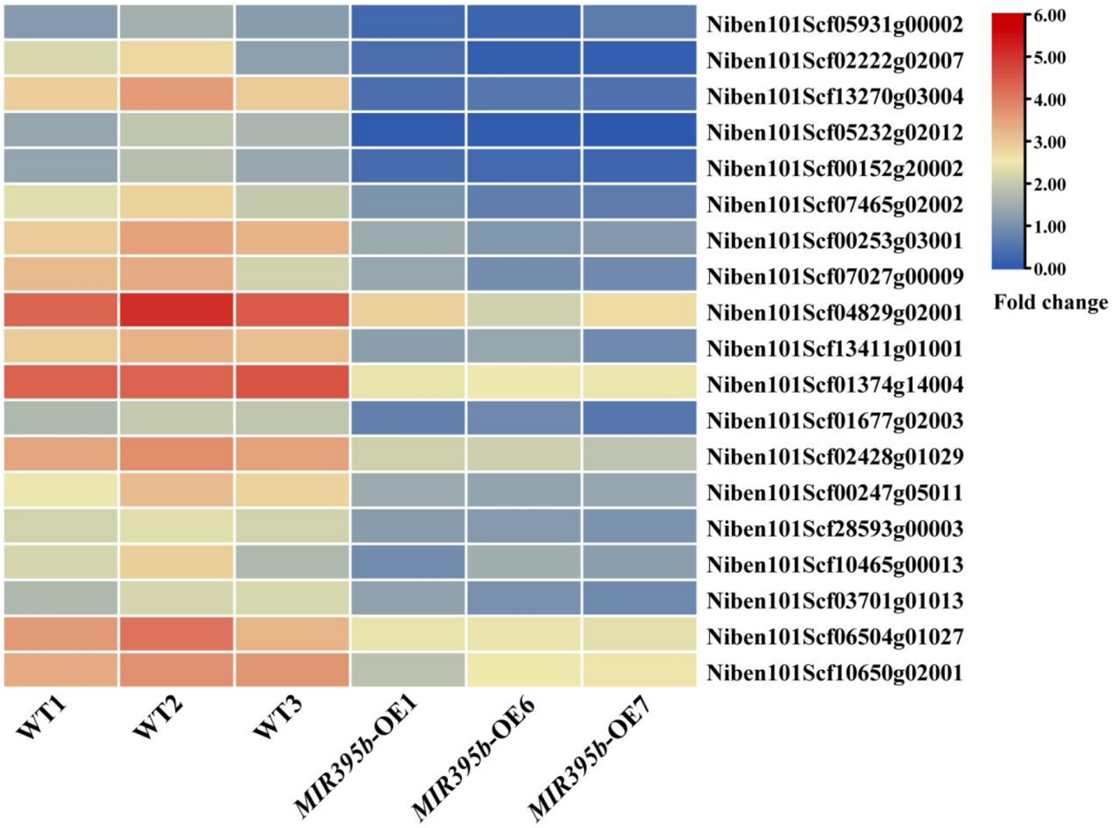

Supplementary Figure S3. Cluster heatmap analysis of differentially expressed genes between WT and *MIR395b*-OE tobacco lines. Genes that show signaling for auxin and abscisic acid (ABA) hormones.

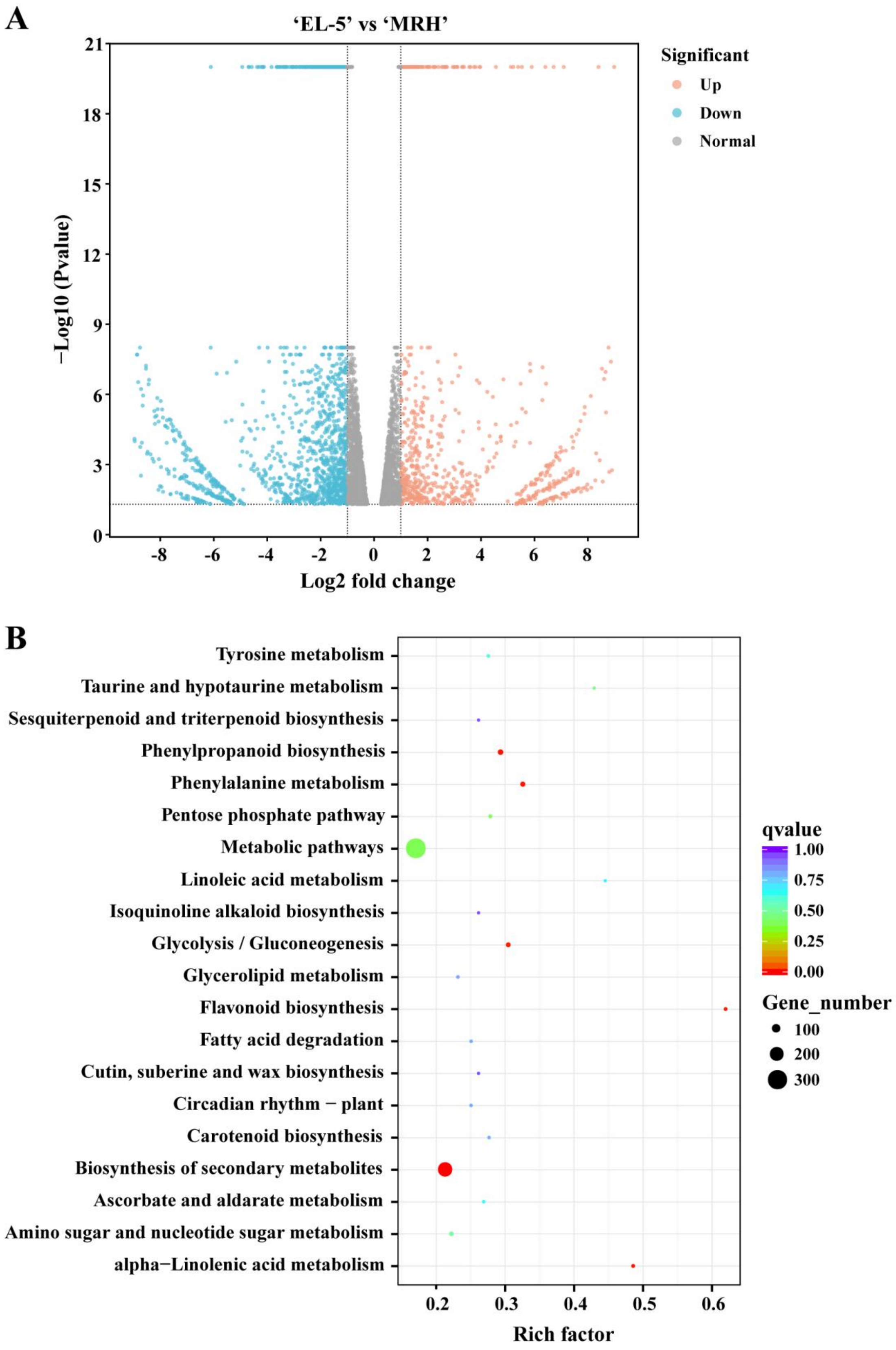

Supplementary Figure S4. RNA transcriptome analysis of 'EL-5' and 'MRH'. **A)** Number of down- and upregulated DEGs between 'EL-5' and 'MRH'. **B)** Significantly enriched KEGG pathways in 'EL-5' and 'MRH'. There were three biological replicates (n = 3) for RNA sequencing.

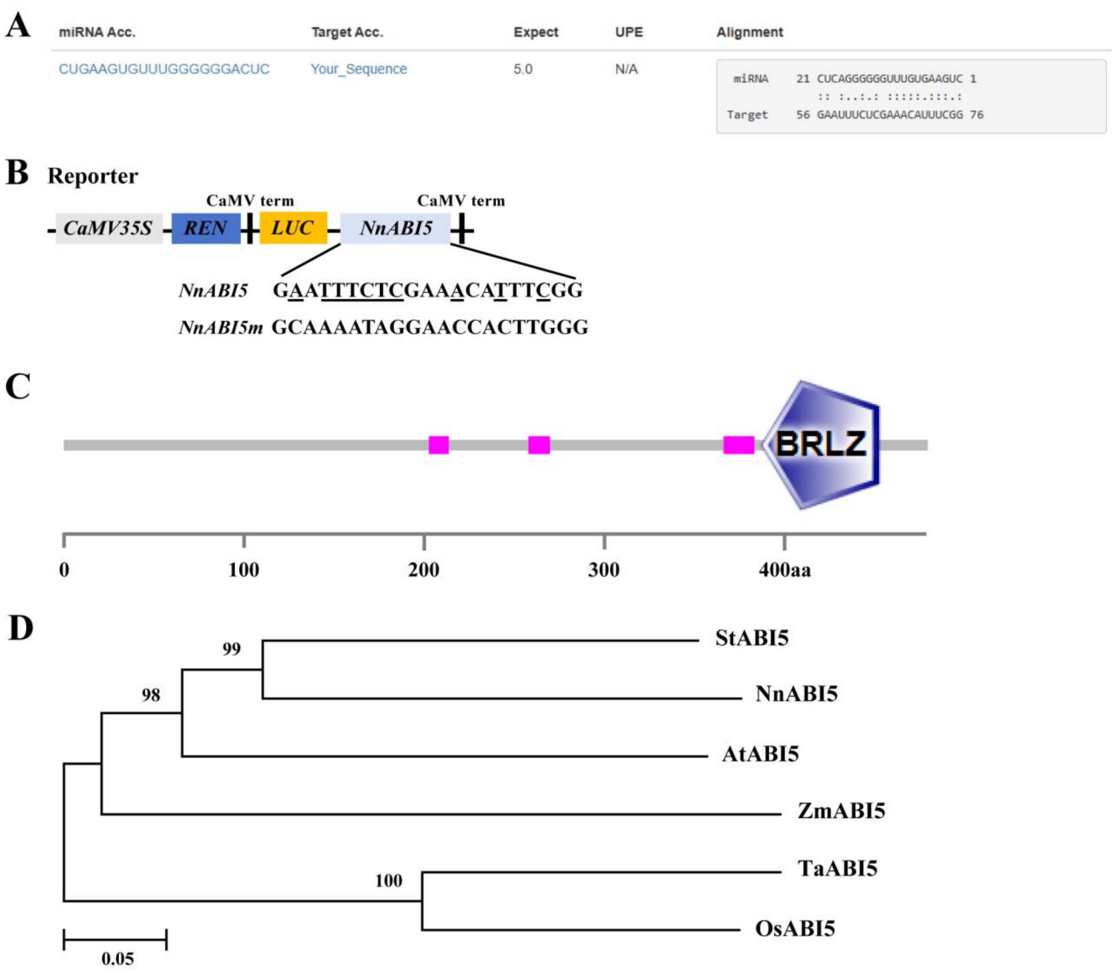

Supplementary Figure S5. Characterization of NnABI5. **A)** Predicted miR395b target genes by psRNATarget (https://www.zhaolab.org/psRNATarget/). **B)** Diagrams of the *LUC-NnABI5m* and *LUC-NnABI5m* constructs. **C)** Conserved domain analysis of the NnABI5 protein by SMART prediction (https://smart.embl.de/). Scale bar represents the protein length. **D)** Phylogenetic analysis of NnABI5. Bootstrap values indicate the confidence of each branch, and the scale indicates the branch length. The scale bar represents 0.05 substitutions per site.

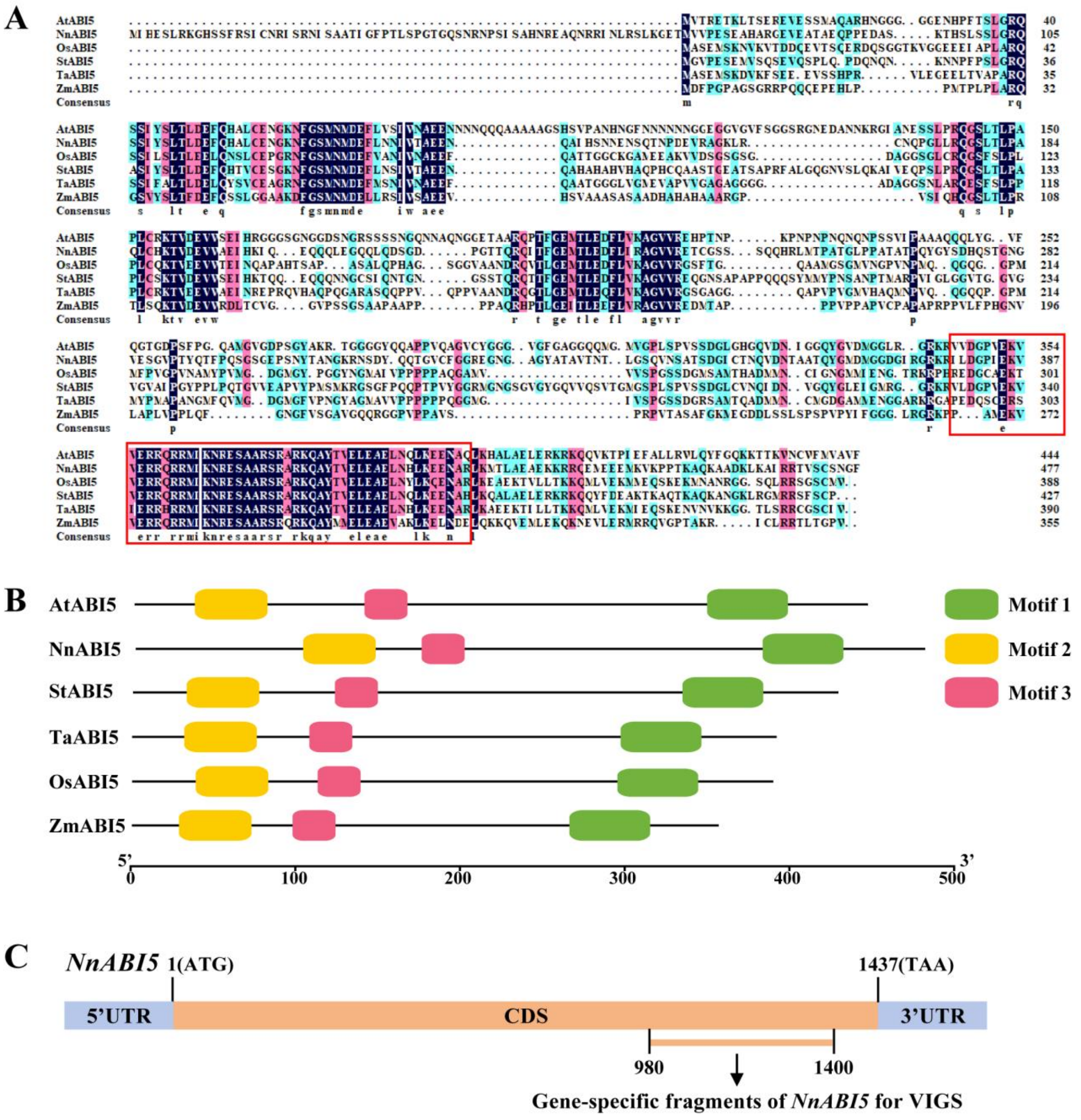

Supplementary Figure S6. Bioinformatics analysis of ABI5. **A)** Alignment of putative amino acid sequences of the NnABI5 protein with five other species. The conserved ABI5 domain is shown using a red frame, and amino acids that are identical in 100% of aligned sequences are shown with the same color background. The amino acids with blue, red, and green backgrounds respectively represents 100%, 80%, and 50% of identity. **B)** Motif compositions of the ABI5 protein. The motifs are numbered 1–3 and displayed in different colored boxes. **C)** Schematic representation of the gene-specific fragments of *NnABI5* for construction of the *NnABI5*-TRV vector.

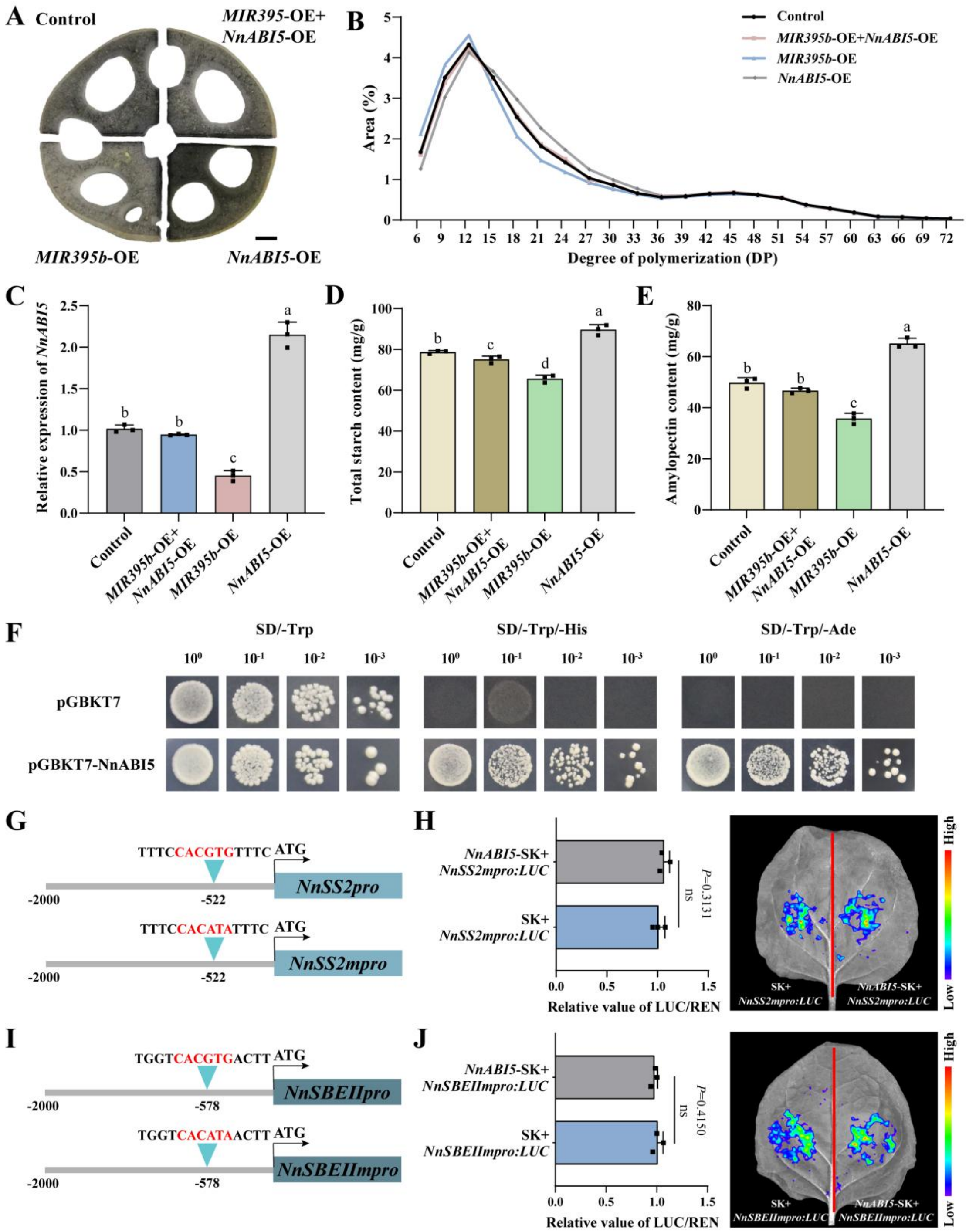

Supplementary Figure S7. NnABI5 positively regulates starch biosynthesis in lotus roots and is inhibited by miR395b. **A)** Starch detection by iodine staining in lotus roots. The scale bar represents 1 cm. Images were digitally extracted for comparison. The experiments were performed independently twice with similar results, and one representative result is shown. **B)** Differences in the distribution of amylopectin chain lengths between the control, *MIR395b*-OE, *NnABI5*-OE, and *MIR395b*-OE + *NnABI5*-OE. Data are shown as means ± SD (n = 3). **C)** Relative transcript levels of *NnABI5* in the lotus roots of the control, *MIR395b*-OE, *NnABI5*-OE, and *MIR395b*-OE + *NnABI5*-OE. Data are shown as means ± SD (n = 3). **D and E)** Starch content analysis of levels in

lotus roots of the control, *MIR395b*-OE, *NnABI5*-OE, and *MIR395b*-OE + *NnABI5*-OE. (D) Total starch content, and (E) the amylopectin content. Data are shown as means ± SD (n = 3). Different lowercase letters (in (C), (D), and (E)) indicate significant differences according to oneway ANOVA with Tukey's multiple comparisons test ($P < 0.05$). **F)** Verification of the self-activation mechanism of the NnABI5 transcription factor in yeast. **G)** Schematic diagram of normal and mutant *NnSS2* promoter. Red letters represent the binding motifs, and their corresponding mutation motifs. **H)** Dual-luciferase assays between NnABI5 and the mutant *NnSS2* promoter. Data are shown as means ± SD (n = 3). Statistical analysis was performed using a one-way ANOVA. Significant levels are denoted by asterisks (two-sided Student's *t*-test; ns, no significant difference). **I)** Schematic diagram of normal and mutant *NnSBEII* promoter. Red letters represent the binding motifs, and their corresponding mutation motifs. **J)** Dual-luciferase assays between NnABI5 and the mutant *NnSBEII* promoter. Data are shown as means ± SD (n = 3). Statistical analysis was performed using a one-way ANOVA. Significant levels are denoted by asterisks (two-sided Student's *t*-test; ns, no significant difference).
