# Supplementary figures and images for: The miR395b–ABI5 module regulates amylopectin branching and biosynthesis and affects lotus root quality
Source: Plant Physiol. 2025 Nov 5;199(3):kiaf554. doi: 10.1093/plphys/kiaf554 (PMC12610401; doi:10.1093/plphys/kiaf554)

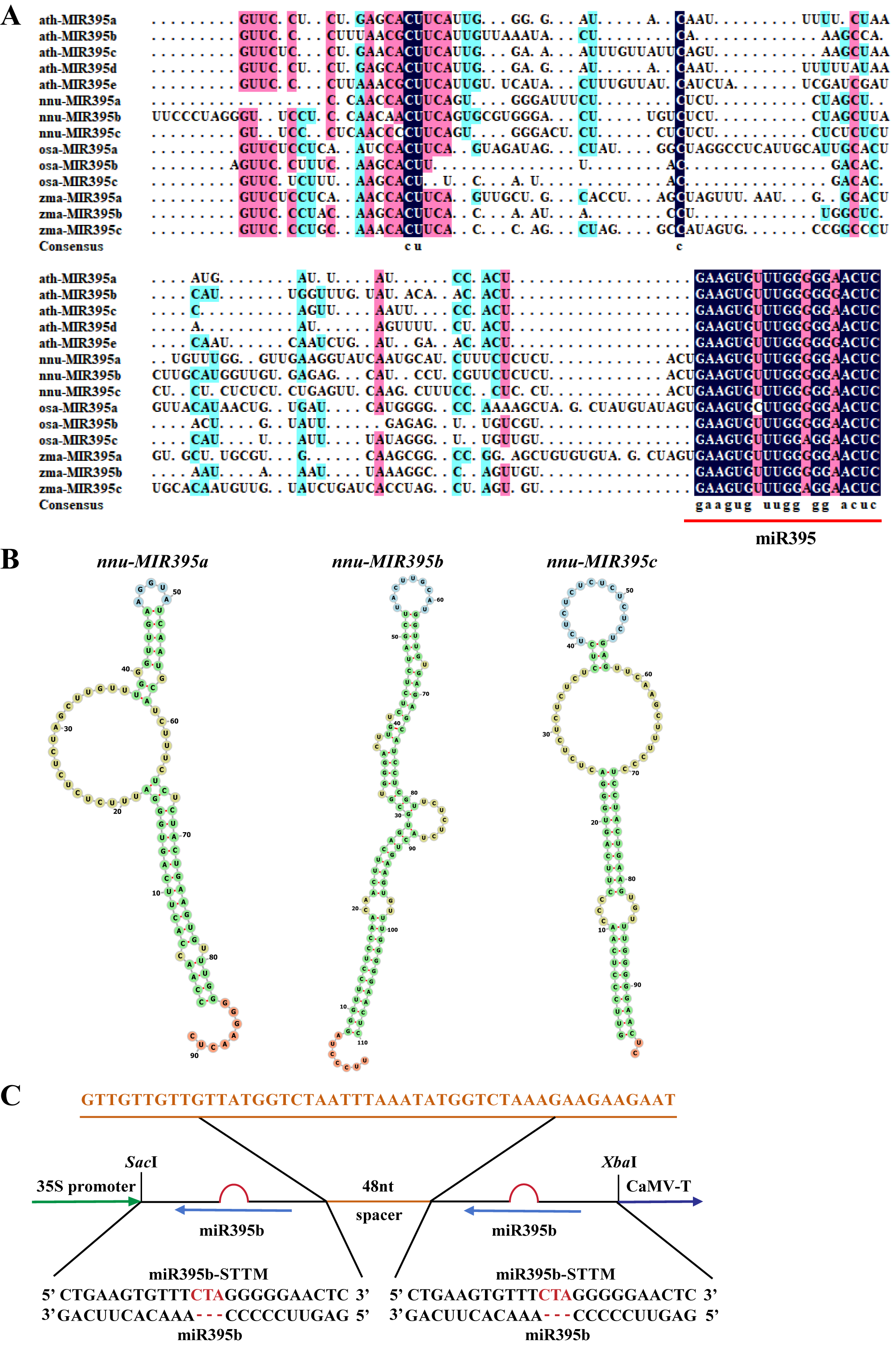

Supplement: kiaf554_Supplementary_Data [file kiaf554_supplementary_data.zip › Supplementary Figure S1.tif]

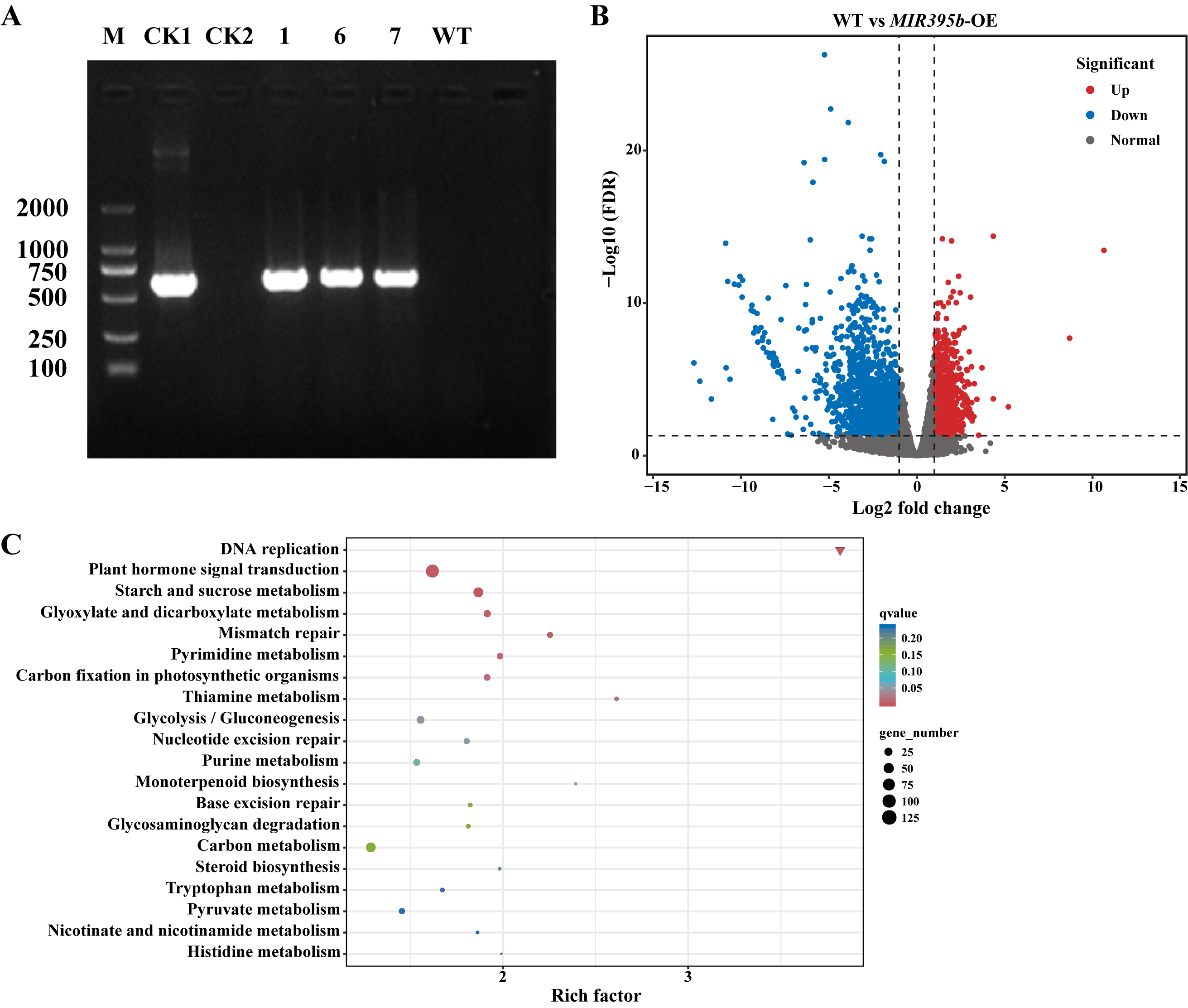

Supplement: kiaf554_Supplementary_Data [file kiaf554_supplementary_data.zip › Supplementary Figure S2.tif]

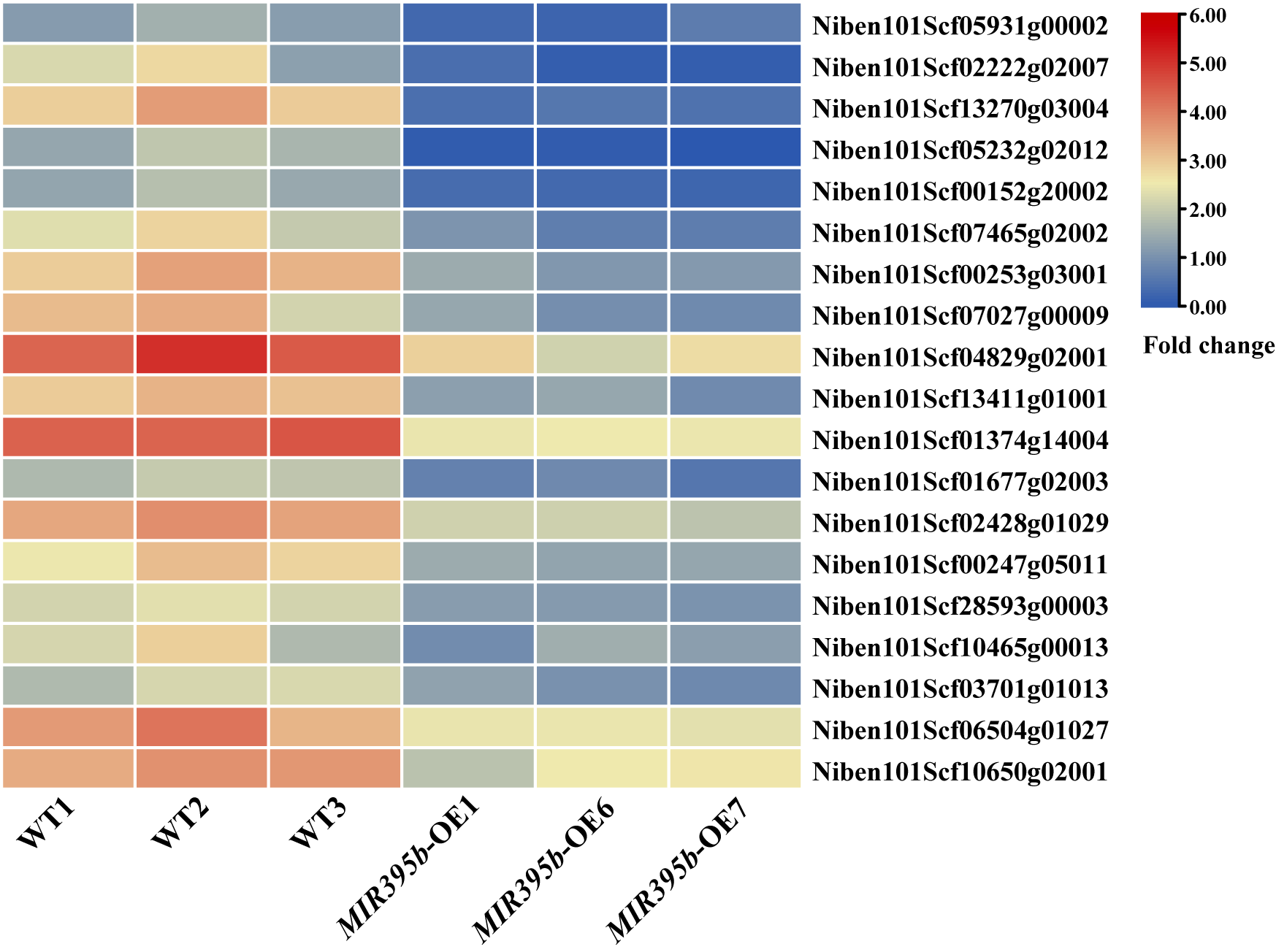

Supplement: kiaf554_Supplementary_Data [file kiaf554_supplementary_data.zip › Supplementary Figure S3.tif]

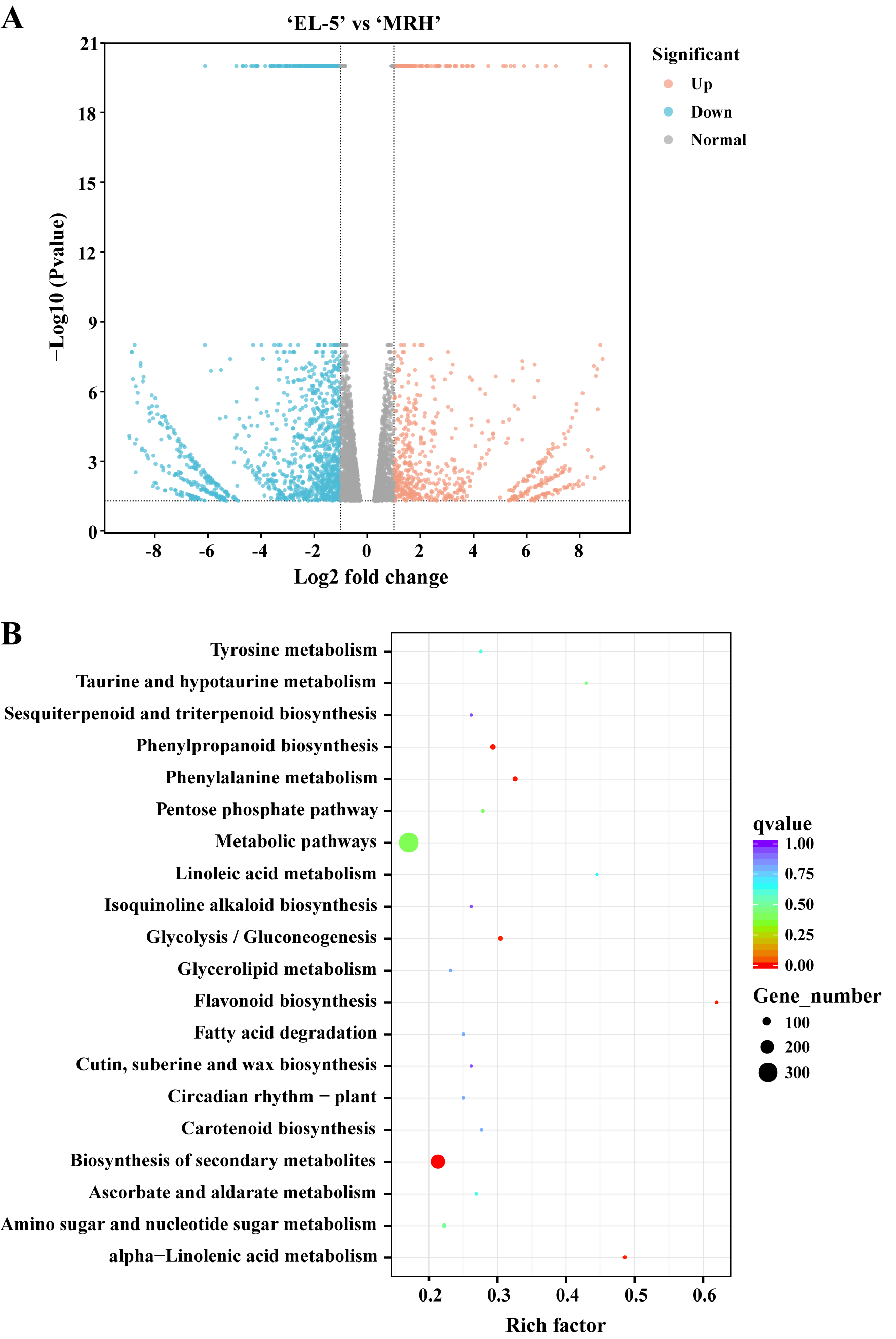

Supplement: kiaf554_Supplementary_Data [file kiaf554_supplementary_data.zip › Supplementary Figure S4.tif]

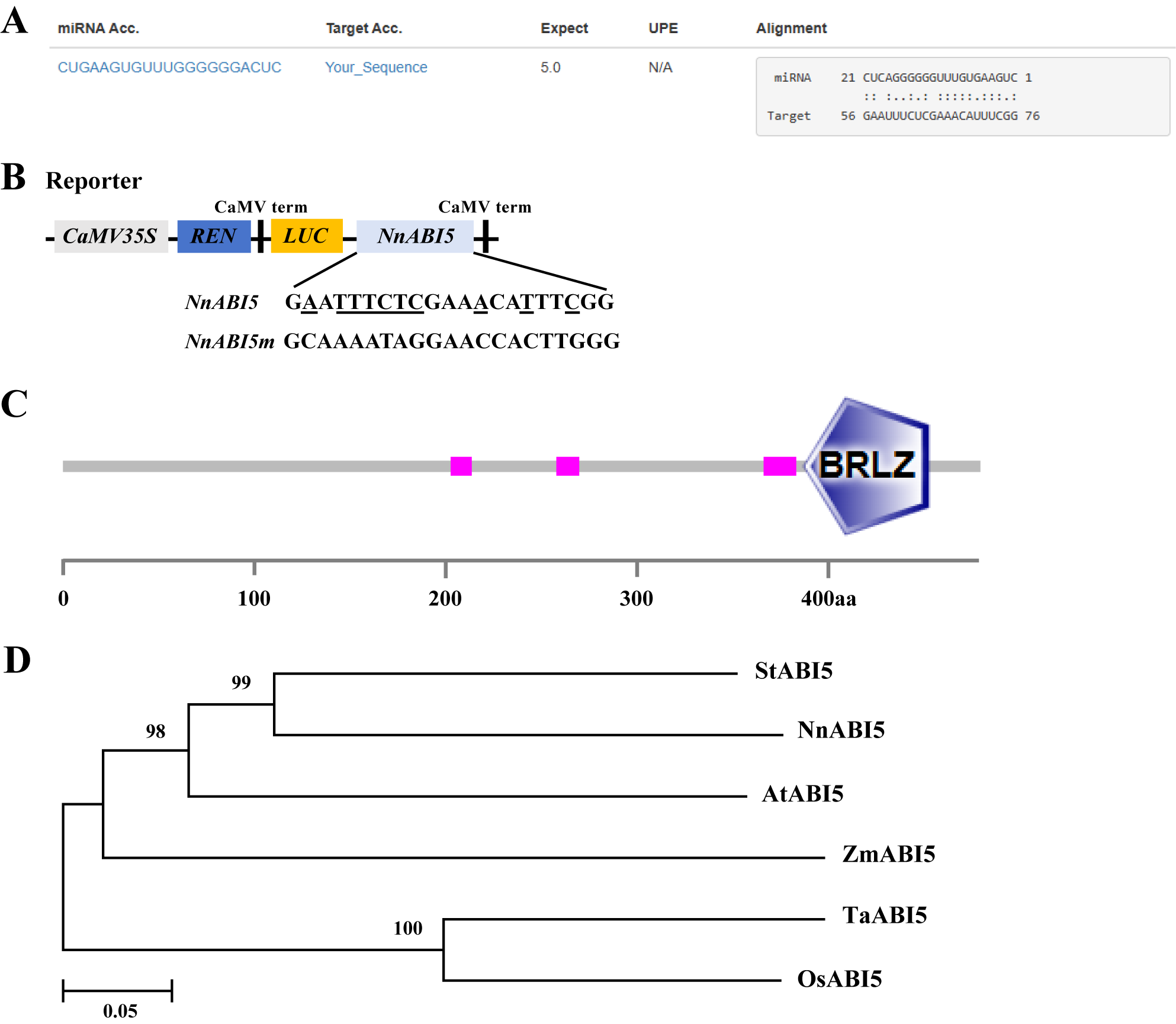

Supplement: kiaf554_Supplementary_Data [file kiaf554_supplementary_data.zip › Supplementary Figure S5.tif]

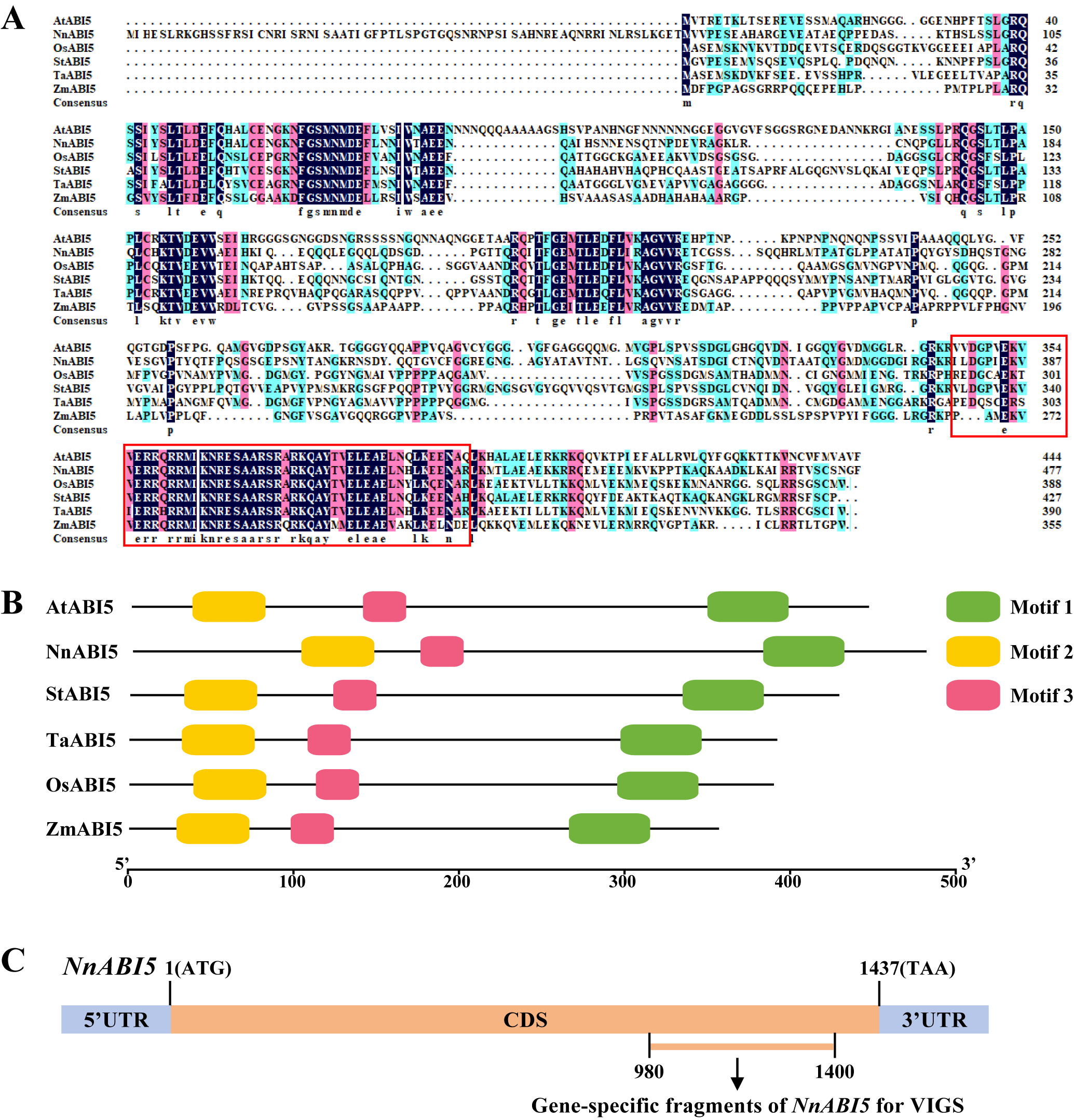

Supplement: kiaf554_Supplementary_Data [file kiaf554_supplementary_data.zip › Supplementary Figure S6.tif]

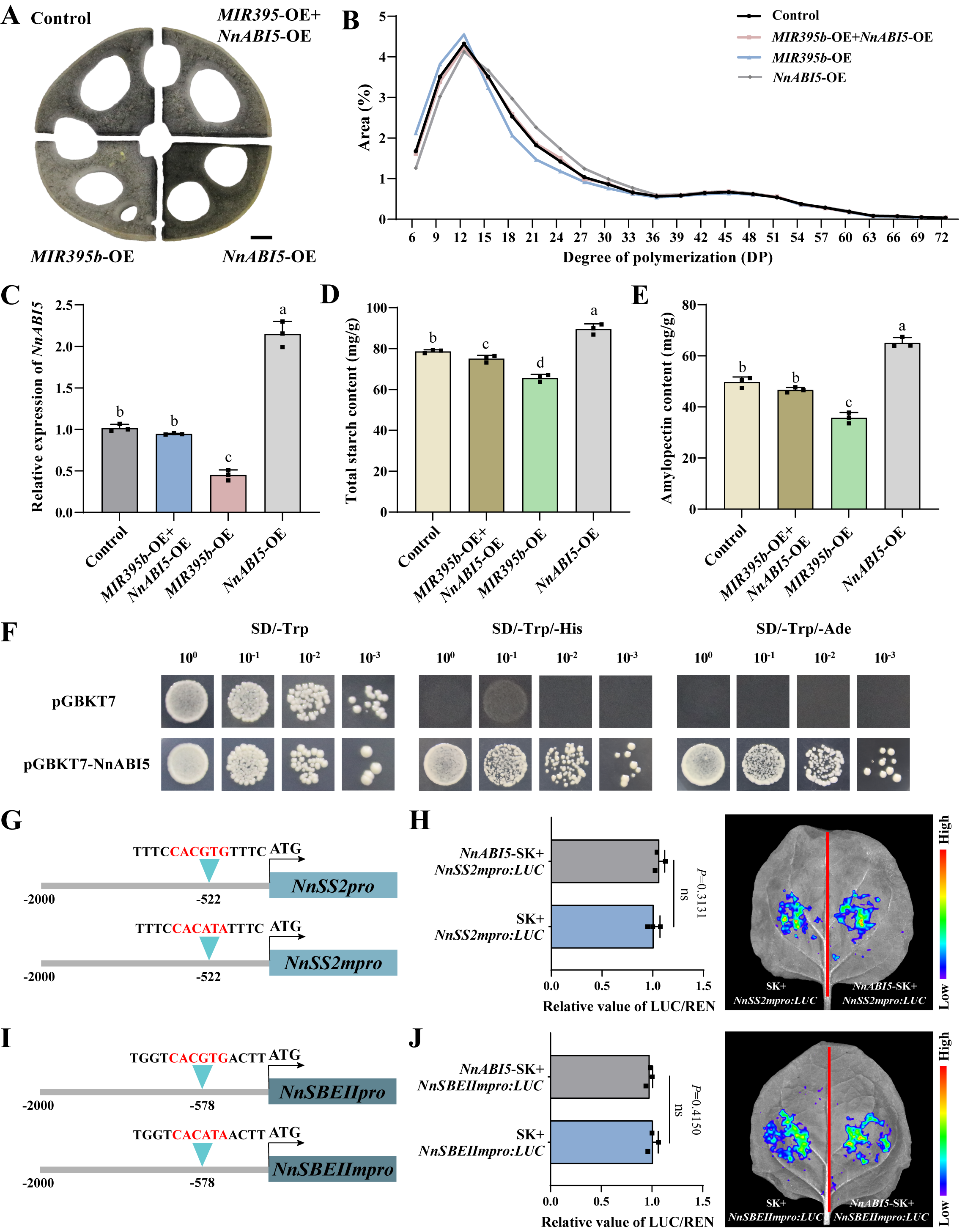

Supplement: kiaf554_Supplementary_Data [file kiaf554_supplementary_data.zip › Supplementary Figure S7.tif]
